# Beyond Mucosal Healing: Quantitative Transmural Phenotyping to Assess Therapeutic Response and Support Precision Treatment in Crohn’s Disease—A Narrative Review

**DOI:** 10.3390/jcm15218696

**Published:** 2026-11-07

**Authors:** Sandro Mereu, Andrea Michielan, Giacomo Sica, Pier Paolo Mainenti, Armando Gabbrielli, Salvatore A. Masala, Mariano Scaglione, Maria Pina Dore, Elettra Merola

**Affiliations:** 1 Department of Medicine, Surgery and Pharmacy, University of Sassari, Viale San Pietro 43, 07100 Sassari, Italy; s.mereu6@studenti.uniss.it (S.M.); samasala@uniss.it (S.A.M.); mpdore@uniss.it (M.P.D.); emerola@uniss.it (E.M.); 2 Gastroenterology and Digestive Endoscopy Unit, Azienda Sanitaria Universitaria Integrata del Trentino (ASUIT), 38122 Trento, Italy; andrea.michielan@asuit.tn.it (A.M.); armando.gabbrielli@unitn.it (A.G.); 3 UOC of Radiology, Ospedale Sacro Cuore di Gesù, Fatebenefratelli, 82100 Benevento, Italy; gsica@sirm.org; 4 Department of Life Sciences, Health, and Healthcare Professions, Link Campus University, 00100 Rome, Italy; 5 Consiglio Nazionale delle Ricerche (CNR), Institute for Biostructure and Bioimages, 80145 Naples, Italy; pierpamainenti@hotmail.com

**Keywords:** Crohn’s disease, transmural phenotyping, imaging biomarkers, intestinal ultrasound, magnetic resonance enterography, computed tomography enterography, radiomics, artificial intelligence

## Abstract

Crohn’s disease (CD) is a transmural disorder in which mucosal healing may coexist with persistent mural, mesenteric, or penetrating disease. Cross-sectional imaging can therefore extend assessment beyond lesion detection toward quantitative transmural phenotyping, using imaging-derived markers to characterize inflammatory activity, structural and fibrotic burden, extramural involvement, and longitudinal treatment response. This narrative review synthesizes recent evidence on intestinal ultrasound (IUS), including contrast-enhanced ultrasound and elastography, magnetic resonance enterography (MRE), computed tomography enterography (CTE), multienergy CT, radiomics, and artificial intelligence (AI). IUS and MRE currently provide the most clinically mature approaches for serial assessment through bowel wall thickness, vascularity, composite activity indices, and measures of transmural response or healing. Conventional CTE remains important for structural and penetrating complications but is primarily morphologic and semi-quantitative. Multienergy CT introduces material-specific biomarkers, particularly iodine-based measures of inflammatory activity, whereas radiomics and AI remain largely investigational. Across modalities, persistent imaging abnormalities after treatment may provide prognostic information beyond mucosal assessment, but definitions, acquisition protocols, thresholds, and validation strategies remain heterogeneous. Quantitative transmural phenotyping may complement clinical, biochemical, endoscopic, and histological assessment, but standardization, multicenter validation, and prospective imaging-guided trials are required before routine precision-management implementation.

## 1. Introduction

Inflammatory bowel diseases, including ulcerative colitis and Crohn’s disease (CD), are chronic immune-mediated conditions marked by flares and periods of quiescence, with a rising prevalence in Western countries. 

CD can affect any segment of the gastrointestinal tract and is characterized by a segmental, transmural pattern of inflammation that, over time, may lead to structural bowel damage, strictures, penetrating complications, and surgery [1]. In recent years, treat-to-target strategies have moved therapeutic goals beyond symptom control, making objective disease control and mucosal healing central to clinical management. However, in CD, no single measure is sufficient: symptoms, biomarkers, colonoscopy, and cross-sectional imaging each describe a different aspect of the disease. This is particularly relevant when mucosal improvement coexists with persistent mural or extramural abnormalities [2,3,4,5,6].

Current standards for measuring disease activity and severity include clinical scores, such as the Crohn’s Disease Activity Index and the Harvey-Bradshaw Index [7,8]. These tools remain useful because they make symptoms measurable and comparable, both in clinical trials and during follow-up. However, clinical scores and symptom-based outcomes cannot define the inflammatory burden, transmural involvement, or structural damage of CD. Biochemical biomarkers are also commonly used in routine CD assessment. C-reactive protein (CRP) provides systemic inflammatory information, whereas fecal calprotectin (FC) is more closely related to intestinal mucosal inflammation and is frequently used for non-invasive monitoring. Their main limitation is anatomical: CRP may be normal despite active CD or elevated for extraintestinal reasons, and FC mainly reflects mucosal activity and may be less reliable in isolated small-bowel disease.

Regarding endoscopic assessment, ileocolonoscopy allows direct visualization of the terminal ileum and colonic mucosa, as well as tissue sampling for histological evaluation [6,9]. Endoscopic assessment is supported by validated scoring systems designed to quantify mucosal disease activity (e.g., the Crohn’s Disease Endoscopic Index of Severity, CDEIS; the Simple Endoscopic Score for Crohn’s Disease, SES-CD) [10,11]. 

Mucosal healing has become a major therapeutic target in CD, and it is incorporated into treat-to-target strategies [12]. At the same time, endoscopy is subject to certain limitations. First, ileocolonoscopy is anatomically limited to the colon and terminal ileum, whereas CD may involve any segment of the gastrointestinal tract, including proximal small-bowel segments beyond the reach of conventional endoscopy [9]. In addition, endoscopic improvement does not necessarily indicate resolution of bowel-wall or extramural disease, and patients with persistent imaging abnormalities may have a different clinical course from those with concordant mucosal and transmural improvement [2,3,4].

Small-bowel capsule endoscopy (SBCE) and device-assisted enteroscopy (DAE) complement cross-sectional imaging in the assessment of proximal small-bowel CD. SBCE provides direct visualization of mucosal abnormalities, including subtle lesions that may be missed by cross-sectional imaging, whereas DAE enables targeted biopsies and selected therapeutic interventions. However, these techniques primarily assess mucosal disease and cannot comprehensively characterize transmural inflammation, fibrotic remodeling, or extramural complications. Moreover, capsule retention remains a concern in patients with established CD, particularly in the presence of strictures, requiring appropriate assessment of small-bowel patency. Conversely, IUS, MRE, and CTE provide information on bowel-wall and extramural abnormalities beyond the reach of endoscopic evaluation. These modalities should therefore be considered complementary, with their selection guided by the clinical question and disease phenotype [13].

Cross-sectional imaging allows assessment of the entire bowel wall and surrounding tissues, thereby providing information on transmural inflammation and on structural or penetrating complications such as strictures, fistulas and abscesses, while in follow-up it can evaluate their changes after treatment initiation [9,14]. However, the clinical interpretation of these findings remains limited by the difficulty of translating complex transmural abnormalities into standardized quantitative measures, prompting growing interest in imaging-derived biomarkers that may provide objective measures of disease burden and treatment response.

The aim of this narrative review is to critically synthesize the evidence on imaging-derived quantitative biomarkers of transmural and extramural CD, focusing on their ability to characterize extramural involvement, longitudinal response, inflammatory activity, and structural fibrotic burden. 

## 2. Literature Search and Study Selection

This review adopted a structured narrative approach aimed at providing an up-to-date, clinically focused synthesis of the available evidence. The literature search and selection process prioritized studies relevant to the proposed framework, without seeking the comprehensive identification and inclusion of all potentially eligible publications required for a systematic review. The limitations associated with this methodological approach are acknowledged and discussed below.

A literature search was conducted and subsequently updated on 4 August 2026 to retrieve relevant studies investigating imaging-derived quantitative or semi-quantitative markers of inflammatory activity, transmural response or healing, stricturing or fibrotic burden, mesenteric or extramural involvement, prognosis, or treatment monitoring.

The initial literature search was carried out in PubMed combining Crohn-related terms with imaging and biomarker terms, including intestinal ultrasound (IUS), magnetic resonance enterography (MRE), computed tomography enterography (CTE), multienergy CT, dual-energy CT (DECT), spectral CT, photon-counting CT (PCCT), radiomics, and artificial intelligence (AI). The complete search strategy is reported in the Supplementary Materials. The PubMed search retrieved 828 records. Additionally, the reference lists of eligible articles, relevant reviews, and guidelines were manually screened to identify further pertinent studies not captured by the electronic search. Overall, 67 original studies were selected for inclusion in Supplementary Tables S1–S5.

Eligible articles were English-language human studies in adult CD, priority was given to original studies published from 2021 to 4 August 2026. Older landmark studies, guidelines, consensus statements, and systematic reviews were used to define established concepts, imaging indices, terminology, and reporting standards, but were not included in the main evidence tables. Because this is a narrative review, no formal risk-of-bias assessment or meta-analysis were performed. 

Within the structured narrative-review framework, two authors (S.M. and E.M.) independently evaluated study eligibility and selected relevant publications. Disagreements regarding inclusion were resolved through discussion until consensus was achieved. Methodological information was extracted from the original articles as reported by the study authors.

OpenAI ChatGPT (GPT-6; OpenAI, San Francisco, CA, USA) was used for English-language editing, stylistic refinement, and assistance in preparing schematic figures; the authors independently reviewed and approved all final content and take full responsibility for the manuscript.

## 3. Concept of Quantitative Transmural Phenotyping in CD

CD is a clinically heterogeneous disorder characterized by different phenotypes based on disease location and behavior, ranging from inflammatory phenotype to stricturing and penetrating patterns according to the Montreal classification [15].

Quantitative transmural phenotyping refers to imaging-based characterization and measurement of mural and extramural disease features in relation to the patient’s anatomical phenotype, both at a given time point and longitudinally during follow-up. The measurable features of transmural assessment can be organized into complementary domains: inflammatory activity, structural and fibrotic burden, extramural involvement, and longitudinal response. The main modality-specific biomarkers contributing to these domains are summarized in Table 1.

Active mural inflammation is best understood as a composite imaging pattern. Bowel wall thickness (BWT) is the most widely used quantitative marker in studies of transmural response and healing, but residual thickening alone is difficult to interpret. Its inflammatory meaning depends on associated activity signs, including increased color Doppler signal and altered bowel wall stratification on IUS, mural edema, enhancement, perfusion changes on cross-sectional or contrast-enhanced imaging, and diffusion restriction on MRE. This distinction is relevant during follow-up: a segment that remains thickened but loses vascular, edematous, or enhancing activity may represent a different phenotype from a segment with a persistent inflammatory signal. Quantitative assessment of mural inflammation should therefore combine wall thickness with modality-specific activity markers [16,17].

Imaging may also identify bowel-wall remodeling, luminal narrowing, stiffness and suspected fibrotic change, allowing structural damage to be evaluated beyond obstructive symptoms alone [18]. In stricturing disease, the key issue is whether narrowing is mainly driven by active inflammation, fixed remodeling, or a mixed pattern [19]. This distinction may guide medical optimization or lead to endoscopic or surgical treatment of the strictures, in line with current European Crohn’s and Colitis Organisation (ECCO) guidance and individualized treat-to-target goals [12,20].

Extramural involvement describes the component of CD that extends beyond the bowel wall and includes peri-intestinal and mesenteric abnormalities detectable on cross-sectional imaging. Imaging may identify inflammatory mesenteric fat, creeping-fat-related changes, enlarged lymph nodes, free fluid and other peri-enteric abnormalities [16]. Penetrating complications further expand this domain, since fistulas and abscesses define a more complex anatomical phenotype and may require integrated medical, interventional, or surgical planning. Extramural assessment adds complementary information to mural inflammation by capturing peri-intestinal abnormalities that may persist or evolve during follow-up [18].

**Figure 1:**
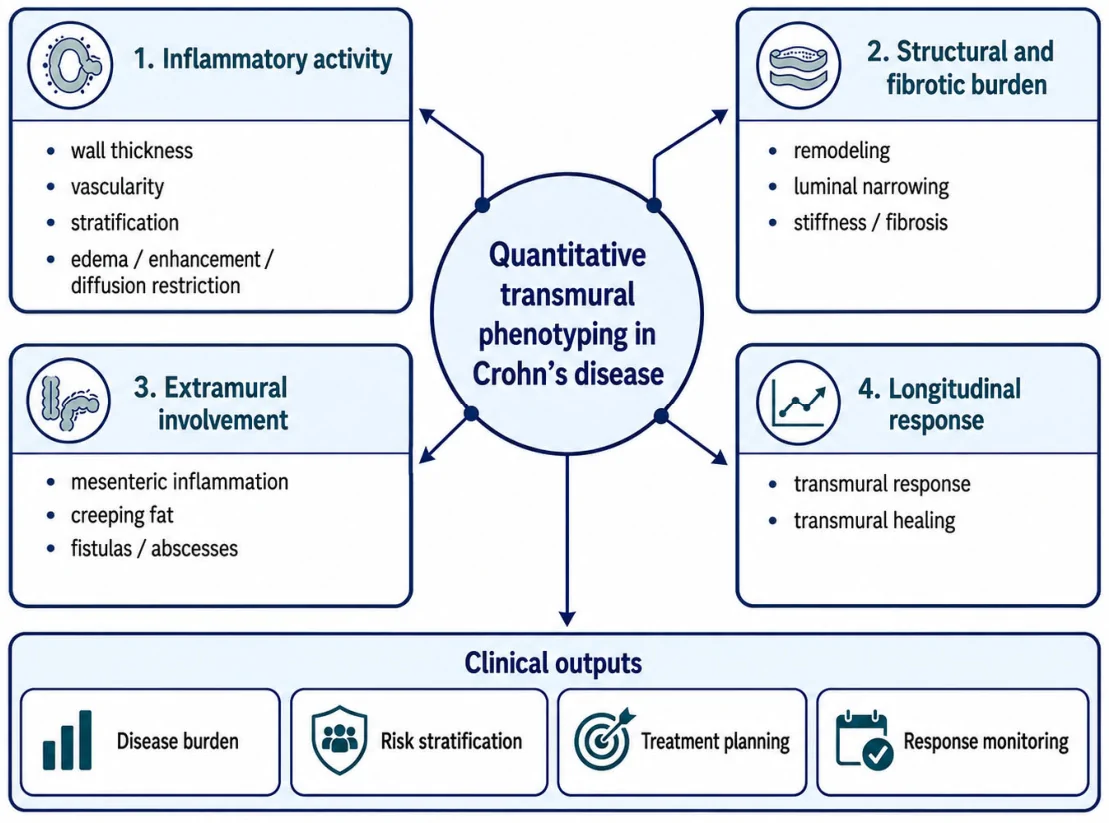
Conceptual framework for quantitative transmural phenotyping in Crohn’s disease. Imaging-derived biomarkers can capture mural, mesenteric, and extramural components of Crohn’s disease, including inflammatory activity, structural and fibrotic burden, complications, and longitudinal treatment response. Quantitative transmural phenotyping combines information from ultrasound, magnetic resonance enterography, computed tomography enterography, multienergy computed tomography, radiomics, and artificial intelligence to complement clinical, biochemical, endoscopic, and histological assessment. Standardization and prospective validation remain necessary before these markers can be used as independent treatment-guiding tools.

Longitudinal response represents the dynamic dimension of transmural phenotyping. The assessment of mural and extramural abnormalities can be used to describe transmural response or, when normalization is more complete, transmural healing. The aim is to reveal the evolution of measurable imaging abnormalities, particularly BWT, vascularity, edema, enhancement, diffusion restriction, and complications. Available definitions of transmural healing remain heterogeneous. In the systematic review by Geyl et al. [4], BWT was the most used parameter, usually with a threshold of ≤ 3 mm, whereas color Doppler signal, contrast enhancement, and absence of complications were incorporated less consistently across studies. The main domains of quantitative transmural phenotyping and their clinical outputs are summarized in Figure 1.

## 4. IUS, CEUS, and Elastography for Quantitative Transmural Phenotyping

IUS is a first-line, repeatable and patient-friendly technique for transmural assessment in CD. It allows direct evaluation of bowel-wall and peri-intestinal abnormalities without ionizing radiation and can be performed during outpatient visits or at the bedside, making it particularly suitable for early reassessment and longitudinal monitoring. Its main parameters include BWT, color Doppler signal, bowel wall stratification (BWS), inflammatory mesenteric fat, lesion length, strictures, prestenotic dilation, fistulas and abscesses. Composite IUS scores, including the Simple Ultrasound Score for Crohn’s Disease (SUS-CD), Bowel Ultrasound Score (BUSS), and International Bowel Ultrasound Segmental Activity Score (IBUS-SAS), have been proposed to standardize segmental assessment of inflammatory activity and are summarized in Table 2.

IUS is already an established tool for monitoring disease activity and therapeutic response in CD, as supported by current ECCO-ESGAR-ESP-IBUS guidelines, which recommend early cross-sectional imaging reassessment following treatment initiation or optimization [6,9]. However, although IUS-defined transmural healing is associated with favorable long-term outcomes, its role as an independent treatment target remains insufficiently validated. In particular, prospective interventional evidence is still needed to determine whether treatment adjustments specifically aimed at achieving transmural healing improve clinical outcomes beyond established treatment targets.

Serial studies have shown that BWT reduction, reduction in color Doppler signal and normalization of mural or mesenteric abnormalities can document early and progressive response to biologic therapy. Transmural healing rates remain lower than partial response rates and vary according to definition, but early IUS improvement has been associated with favorable later sonographic, endoscopic, or clinical outcomes [16,28,29,30]. Prospective data also suggest that baseline and postinduction IUS scores may contribute to risk stratification, particularly when BWT, BUSS or IBUS-SAS are considered together with clinical and biochemical markers [22,31,32]. Additional cohort data support the value of serial IUS changes for outcome assessment and early response prediction [33,34,35]. The main recent original ultrasound-based studies are summarized in Supplementary Table S1 (Supplementary Materials).

A key advantage of IUS is that it does not need to be limited to BWT alone. Mesenteric features, especially inflammatory mesenteric fat and creeping fat, are increasingly recognized as markers of extramural disease burden and adverse outcomes. Inflammatory mesenteric fat has been associated with lower rates of clinical, mucosal and transmural healing after infliximab, whereas recovery of this feature during induction was associated with better 1-year outcomes [36]. In stricturing CD, creeping fat wrapping angle and color Doppler signal may help identify patients at higher risk of surgery [37]. These findings support a broader IUS-based phenotype that integrates mural thickness, color Doppler signal, mesenteric abnormalities and complications rather than relying on a single measurement.

**Figure 2:**
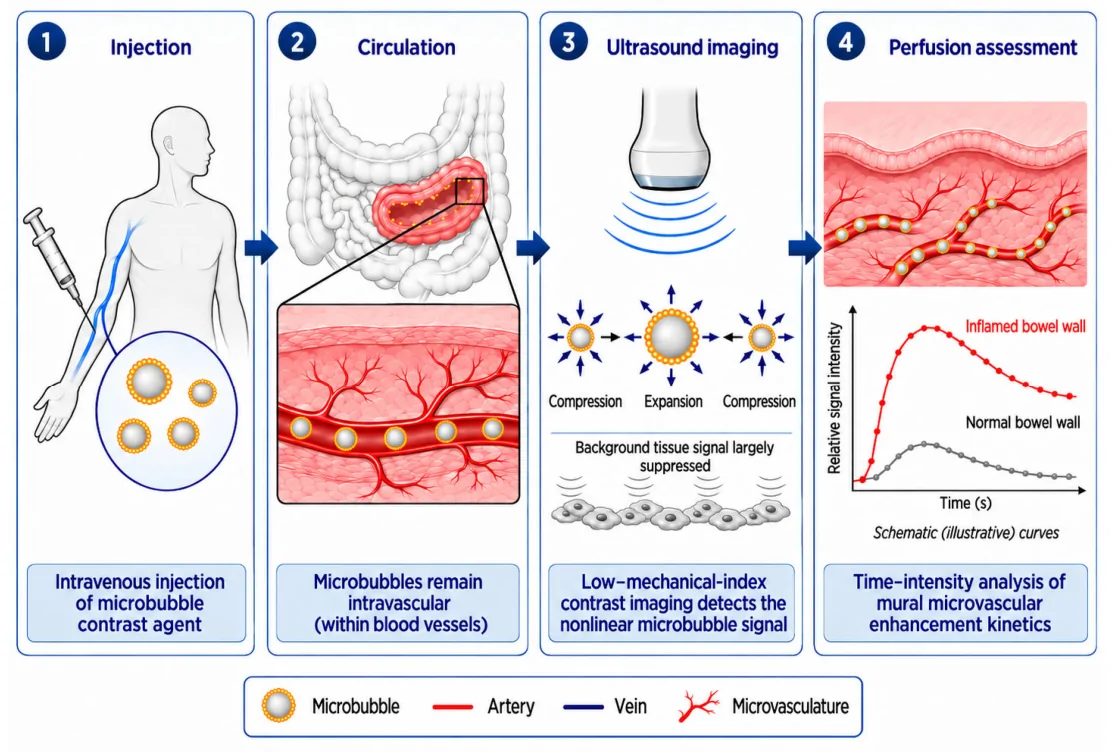
Perfusion assessment by contrast-enhanced ultrasound in Crohn’s disease. Following intravenous administration, gas-filled microbubbles remain confined to the vascular compartment, including the intestinal microcirculation. During low-mechanical-index ultrasound imaging, microbubbles undergo alternating compression and expansion, generating nonlinear acoustic signals that can be selectively detected while background tissue signals are largely suppressed. Time–intensity curve analysis enables quantitative or semi-quantitative characterization of bowel-wall microvascular enhancement, including peak enhancement, time to peak, wash-in, and wash-out kinetics. The curves are schematic and illustrate potential differences in enhancement between inflamed and normal bowel walls rather than patient-derived measurements or validated diagnostic thresholds. CEUS-derived parameters are influenced by acquisition settings, contrast administration, region-of-interest placement, and post-processing methods. Abbreviations: CEUS, contrast-enhanced ultrasound.

Contrast-enhanced ultrasound (CEUS) extends conventional IUS by adding a dynamic assessment of bowel-wall microvascular perfusion. After intravenous administration, microbubbles remain within the vascular compartment and generate contrast-specific signal from flowing blood, allowing real-time evaluation of mural enhancement and time–intensity curve analysis (Figure 2). CEUS-derived parameters describe enhancement magnitude, enhancement timing, cumulative enhancement and wash-in/wash-out kinetics. These parameters are biologically attractive because active transmural inflammation is associated with increased mural vascularity, but their interpretation remains dependent on equipment, acquisition settings, contrast protocol, region of interest (ROI) placement, motion correction and post-processing software. Available studies suggest that CEUS can complement conventional IUS for activity assessment, but do not support its use as a standalone tool to distinguish inflammatory, fibrotic and mixed strictures [38,39]. Multimodal ultrasound approaches combining conventional IUS with CEUS and elastography have also been explored for response prediction [40].

Elastography aims to quantify bowel-wall stiffness and is therefore particularly attractive in stricturing CD. Increased stiffness may reflect fibrosis, muscular remodeling, chronic structural change, active inflammation-related edema, bowel tension, or a mixed process. For this reason, elastography should not be interpreted as a direct measure of fibrosis, but as a complementary stiffness biomarker that may help characterize stricturing disease when integrated with conventional IUS, CEUS, MRE or CTE findings. Early studies have evaluated elastography-derived stiffness in relation to MRE features, stricture biomechanics, longitudinal treatment-related changes, future behavior progression, and stenosis-related parameters, but histological validation and standardized cut-offs remain limited [41,42,43,44,45].

Taken together, ultrasound-based phenotyping is currently most robust for BWT, color Doppler signal and composite IUS scores, whereas CEUS, mesenteric fat assessment and elastography remain complementary tools requiring further standardization and prospective validation.

## 5. MRE: Quantitative Transmural and Extramural Assessment

MRE is a comprehensive cross-sectional modality for quantitative transmural phenotyping in CD because it assesses the bowel wall, mesentery, disease extent, strictures and penetrating complications without ionizing radiation. This is particularly relevant in a chronic disease requiring repeated imaging over time. MRE translates mural and extramural abnormalities into quantitative or semi-quantitative features, including BWT, mural edema, ulceration, enhancement, diffusion restriction, apparent diffusion coefficient (ADC), mesenteric inflammatory changes, luminal narrowing, stricture length and upstream dilation. These features can be integrated into activity and response indices, summarized in Table 2, and used to assess treatment-related change, residual disease burden and stricturing complications.

MRE activity indices are responsive to treatment-related change and provide standardized endpoints for clinical trials and longitudinal assessment. In paired pretreatment and post-treatment examinations, the Magnetic Resonance Index of Activity (MaRIA), simplified MaRIA (sMaRIA), London index and London extended index all showed moderate-to-large responsiveness, with the highest standardized effect size for sMaRIA [46]. Non-contrast approaches are also being explored, particularly to reduce gadolinium exposure in repeated monitoring. A modified sMaRIA incorporating diffusion-weighted categories showed diagnostic performance for active inflammation comparable to contrast-enhanced sMaRIA and better than T2-only sMaRIA [17]. Recent original MRE studies are summarized in Supplementary Table S2 (Supplementary Materials).

Beyond activity measurement, MRE-based transmural response and healing may provide prognostic information. In the DEVISE-CD cohorts, the C-score was developed to define transmural healing and partial transmural response; these endpoints were associated with lower risk of bowel damage progression and better 1-year corticosteroid-free clinical and biochemical remission [27]. In patients starting biologic therapy, early initiation within 12 mo of diagnosis was associated with higher rates of transmural healing, and this endpoint was independently associated with lower risks of bowel damage progression, surgery and treatment escalation [47]. Post-treatment residual abnormalities may be especially informative: MRE reassessment at week 46 after initiation of biological therapy showed that persistent severe inflammatory lesions (MaRIA ≥ 11), penetrating lesions, creeping fat, and stricturing or penetrating complications were associated with subsequent adverse clinical outcomes over a 2-year follow-up period [48].

MRE is also central to stricturing and fibrostenotic CD, where treatment decisions require estimation of inflammatory activity and structural burden. Reproducible MRE features such as stricture length, maximal wall thickness and upstream dilation have been associated with stricture severity and may serve as standardized endpoints for fibrostenosing disease trials [49]. Histology-matched studies suggest that multiparametric MRE may help characterize fibrotic burden, but inflammation, fibrosis and muscular remodeling frequently coexist within the same stricture, limiting the possibility of direct fibrosis quantification [50]. Recent prospective data also indicate that strictures may improve after drug therapy, supporting the role of serial imaging to document structural response rather than assuming all strictures are fixed lesions [51].

Prognosis prediction remains less established than response assessment. In METRIC-EF, adding baseline MRE scores to clinical predictors did not improve prediction of disabling disease over 5 years [52]. Functional magnetic resonance imaging (MRI approaches have also shown limitations: early changes in cine-MRI motility and diffusion-weighted imaging (DWI)-derived parameters did not reliably predict 1-year response or remission beyond conventional biomarkers in the MOTILITY study and DWI substudy [53,54]. These findings suggest that MRE is more mature for documenting disease burden and treatment-related change than for standalone baseline prediction of long-term therapeutic success.

Overall, MRE provides a robust multiparametric approach to quantitative transmural phenotyping, with the strongest evidence currently supporting response assessment, post-treatment prognostic stratification and characterization of stricturing disease. Limitations include cost, availability, bowel preparation, protocol heterogeneity, gadolinium exposure in some protocols, ADC standardization and only fair-to-moderate agreement with IUS for some response definitions [55,56].

### Pelvic MRI and Quantitative Assessment of Perianal Fistulizing Disease

Perianal fistulizing CD represents a distinct manifestation of penetrating disease in which quantitative imaging may complement clinical assessment. Pelvic MRI is the reference imaging modality for evaluating fistula anatomy, tract complexity, inflammatory activity, abscesses, and treatment-related changes. MRI-based indices, including the modified Van Assche index and the Magnetic Resonance Novel Index for Fistula Imaging in Crohn’s Disease (MAGNIFI-CD), have been developed to standardize assessment of fistula severity and response. MAGNIFI-CD incorporates six imaging components and has demonstrated favorable reliability and responsiveness in its initial development and validation [57]. External validation in 65 patients further supported its potential for treatment monitoring, with a reduction of at least 2 points proposed for clinical response and a score ≤6 for remission [58]. However, these cut-offs require further validation and should not be considered universal treatment targets. Recent international consensus recommendations support selected MRI indices for research and clinical trials but do not recommend any individual index for routine clinical decision-making [59]. Although perianal disease fits within the extramural domain of quantitative phenotyping, pelvic MRI requires dedicated acquisition protocols and disease-specific outcome definitions that differ from those used for luminal and mesenteric Crohn’s disease. 

## 6. Standard CT for Quantitative Transmural and Mesenteric Phenotyping

CTE provides rapid cross-sectional assessment of the bowel wall and extramural tissues, combining short acquisition time, high spatial resolution and robust depiction of small-bowel disease and its complications. Its diagnostic performance is broadly comparable to MRE for many CD manifestations, but exposure to ionizing radiation limits its role in repeated monitoring. Accordingly, CTE is generally most useful when rapid assessment is needed, when MRE or IUS are unavailable or contraindicated, or when detailed evaluation of acute or complex complications is required.

Unlike multienergy CT, PCCT or radiomics, conventional CTE does not provide intrinsic tissue-specific quantitative biomarkers. Rather, it contributes to transmural phenotyping through measurable morphologic and enhancement-related features. Some parameters, such as BWT, stricture length, residual luminal diameter, lymph node size and upstream dilation, can be measured directly. Other findings, including mural hyperenhancement, mural stratification, comb sign, mesenteric fat abnormalities, fistulas and abscesses, are visually assessed or semi-quantitative and remain partly reader-dependent. Their interpretation is influenced by bowel distension, acquisition phase, contrast timing and reporting criteria. Therefore, standard CTE should be viewed as a tool for structured morphologic and semi-quantitative phenotyping rather than as a fully quantitative platform for tissue characterization.

Adequate bowel distension is essential for CTE interpretation, because collapsed or underdistended segments may mimic or obscure mural thickening and hyperenhancement. Images are acquired after oral luminal distension and intravenous iodinated contrast administration, usually in the enteric or portal venous phase. Conventional CTE assessment integrates mural thickening, enhancement pattern, mesenteric vascularity, strictures, upstream dilation, fistulas and abscesses, thereby providing complementary information on inflammatory activity, structural burden and extramural involvement. Recent original studies evaluating conventional CTE are summarized in Supplementary Table S3 (Supplementary Materials).

Recent studies support the feasibility of structured CTE assessment of inflammatory activity, although the derived indices should be interpreted as morphologic or semi-quantitative scores rather than objective tissue biomarkers. In a biopsy-correlated study, mural hyperenhancement was mainly associated with neutrophilic inflammation, whereas lymphoplasmacytic inflammation was associated with larger lymph nodes and increased mesenteric fat attenuation, suggesting that individual CTE features may reflect different components of inflammation [60]. However, because histology was based on endoscopic biopsies, these findings cannot be directly extrapolated to full-thickness mural remodeling. Other studies have developed CTE-based activity scores correlated with endoscopic severity. Tong et al. [61] proposed the CT Enterography Index of Activity, based on mural thickness, mural stratification and comb sign, which correlated with endoscopic score (CDEIS) and detected ulcerative lesions with good diagnostic performance. Liu et al. [62] developed a multiparametric CTE score that correlated with endoscopic score (SES-CD) and predicted moderate-to-severe and severe endoscopic activity. These data suggest that CTE can standardize morphologic assessment of activity, but not that conventional CTE directly quantifies tissue composition.

CTE is particularly useful for stricturing and mesenteric phenotyping, where anatomy and luminal geometry are clinically relevant. Recent Society of Abdominal Radiology consensus recommendations define small-bowel stricture on CTE or MRE using wall thickening, luminal narrowing and upstream dilation, emphasizing the need for standardized cross-sectional criteria [18]. Choi et al. suggest that a prestenotic dilation threshold of 2.5 cm may identify additional clinically relevant strictures associated with higher risks of emergency department visits, small-bowel surgery, symptomatic obstruction and penetrating progression [63]. These findings reinforce the value of CTE-derived structural measurements, but also highlight that they describe luminal and morphologic consequences of disease rather than the underlying inflammatory, fibrotic or muscular components of the stricture.

Mesenteric abnormalities may add information to the fibrostenosing phenotype. The mesenteric creeping fat index (MCFI) is a CTE-based semi-quantitative measure of circumferential fat wrapping and has been correlated with macroscopic creeping fat and histological fibrosis severity in surgical cohorts [64]. A model combining MCFI, mesenteric edema and disease duration also showed performance for distinguishing non-mild from moderate-to-severe fibrosis [65]. These findings support the biological relevance of mesenteric CTE features, but the evidence remains largely surgery-based and should not be interpreted as direct noninvasive fibrosis quantification. Similarly, structural CTE measurements such as BWT, residual luminal diameter and upstream dilation have been associated with surgery-free survival in symptomatic stricturing CD, suggesting prognostic value of luminal geometry and structural burden [66].

CTE can also reveal residual transmural or extramural abnormalities when mucosal control appears adequate. Low-dose CTE studies indicate that transmural healing is less frequent than endoscopic healing, and that residual mesenteric edema, fibrofatty proliferation or active small-bowel inflammation beyond endoscopic reach may identify patients at higher risk of relapse [67,68]. In patients treated with ustekinumab, baseline mesenteric features were also associated with the probability of subsequent transmural healing [69]. These observations support the concept that residual radiologic abnormalities may carry prognostic information even after mucosal healing, especially when disease extends beyond the reach of ileocolonoscopy.

In this context, conventional CTE is best viewed as a structured morphologic and semi-quantitative tool for selected acute, complex or contraindication-driven scenarios, rather than as an intrinsic quantitative platform for tissue characterization.

## 7. Multienergy CT for Quantitative Tissue Characterization

**Figure 3:**
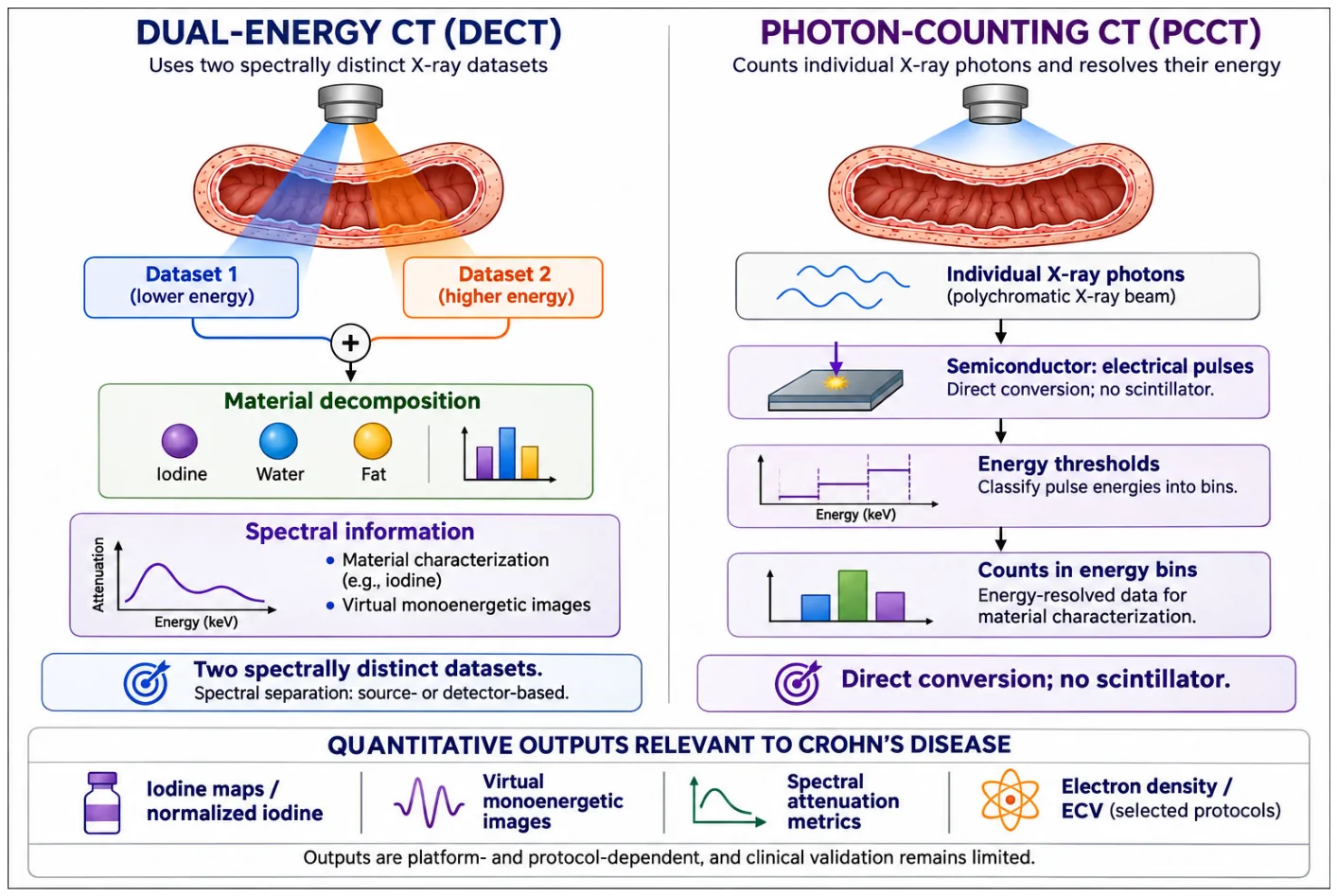
Conceptual comparison between dual-energy CT (DECT) and photon-counting CT (PCCT), and their potential quantitative outputs in Crohn’s disease. DECT acquires two spectrally distinct X-ray datasets, enabling spectral or material decomposition and the generation of derived information such as iodine maps, virtual monoenergetic images, and other spectral attenuation metrics. Spectral separation may be achieved using different source- or detector-based implementations. In contrast, PCCT directly converts individual X-ray photons into electrical signals within a semiconductor detector, without a scintillator, and classifies them according to energy thresholds to generate energy-resolved data. Both techniques may provide quantitative outputs potentially relevant to Crohn’s disease, including iodine-related parameters, virtual monoenergetic images, spectral attenuation metrics, and, in selected protocols, electron density or extracellular volume-related measures. The figure is schematic and is intended to illustrate the underlying technical principles and potential outputs rather than specific acquisition architectures or validated clinical thresholds. Abbreviations: CT, computed tomography; DECT, dual-energy computed tomography; PCCT, photon-counting computed tomography; keV, kiloelectronvolt; ECV, extracellular volume fraction.

Multienergy CT techniques, including DECT, spectral detector CT and PCCT, extend conventional CTE by providing quantitative material-specific information beyond visual assessment of mural hyperenhancement [70]. Conventional CTE can identify a hyperenhancing bowel segment, but conventional attenuation alone provides limited information on the amount and distribution of iodinated contrast within the bowel wall. Spectral CT was developed to extract more quantitative information from the energy-dependent interaction between X-rays and tissues, opening the possibility of moving from visual enhancement toward material-specific biomarkers. The main technical principles and quantitative outputs relevant to CD are summarized in Figure 3.

DECT acquires information from two different X-ray energy spectra and enables post-processing techniques such as virtual monoenergetic imaging and material decomposition. Iodine maps allow contrast enhancement within the bowel wall to be quantified rather than visually judged as increased or decreased. Because contrast uptake reflects, at least in part, tissue perfusion and vascularity, this measurement (iodine density, ID) has been investigated as a quantitative surrogate of inflammatory activity.

PCCT uses different detector technology. Instead of integrating the energy of incoming photons, photon-counting detectors directly register individual photons and classify them according to their energy. This preserves spectral information at the detector level, reduces electronic noise and allows smaller detector elements, with potential gains in spatial resolution, contrast resolution and dose efficiency. For bowel imaging, these properties are appealing because the target is a wall only a few millimeters thick, in which partial-volume effects and heterogeneous enhancement can substantially influence quantitative measurements.

The most frequently investigated parameters include ID, normalized ID (NID), virtual monoenergetic attenuation, spectral curve slope, dual-energy index, electron density and extracellular volume fraction (ECV). These measurements aim to quantify iodine uptake, perfusion-related changes and tissue composition. However, values remain dependent on scanner platform, acquisition phase, contrast protocol, post-processing software, normalization method and ROI strategy. The main recent original studies evaluating multienergy CT are summarized in Supplementary Table S4 (Supplementary Materials).

Iodine-based measurements are currently the most mature multienergy CT biomarkers for inflammatory activity. Histopathology- and endoscopy-correlated studies indicate that ID or NID can distinguish active from inactive inflammation and may help grade inflammatory severity [71,72,73,74,75,76]. However, reported thresholds vary considerably. Mean aorta-normalized mucosal iodine density cut-offs of 20% and 24.6% were identified for histologically and endoscopically active inflammation, respectively [71,72]. Zhou et al. reported normalized iodine concentration (NIC) thresholds of 0.245 in the arterial phase and 0.522 in the venous phase for histological activity [73], whereas Zhu et al. identified an NIC threshold of 0.448 for distinguishing mucosal healing from persistent ulcerative inflammation after infliximab treatment [76]. Regarding absolute iodine density, a threshold of 1.78 mg/mL was reported for endoscopically active inflammation using DECT [72], while preliminary PCCTE data suggested thresholds of 2.7 mg/mL for active inflammation and 3.4 mg/mL for distinguishing moderate-to-severe from mild inflammation [75]. These study-specific values reflect differences in acquisition phases, normalization methods, imaging platforms, and reference standards and should not be interpreted as interchangeable or universally validated clinical thresholds.

Multienergy CT may also contribute to post-treatment assessment and mesenteric phenotyping. After infliximab therapy, iodine measurements differentiated mucosal healing from persistent ulcerative inflammation, but fully healed bowel-wall segments could be difficult to measure because normalization limited ROI placement [76]. ID also appears to vary across morphologic phenotypes, suggesting that iodine uptake primarily reflects inflammatory burden within a phenotype rather than structural complexity itself [77]. Spectral parameters of creeping fat and mesenteric fat have been associated with disease activity, indicating that quantitative assessment of the mesenteric compartment may complement mural measurements [78,79].

Fibrosis characterization remains less mature. Electron density, monoenergetic attenuation and extracellular volume fraction have shown associations with histological fibrosis in small surgical or stricturing cohorts, but performance is influenced by inflammatory severity and the coexistence of fibrosis, inflammation and muscular remodeling [80,81]. Technical PCCT optimization studies further support the feasibility of spectral bowel imaging, but do not yet establish clinical outcome utility in CD [82].

At present, the strongest evidence for multienergy CT supports iodine-based assessment of active inflammation, whereas fibrosis characterization, treatment monitoring and PCCT applications remain promising but preliminary.

## 8. Radiomics and Artificial Intelligence for Quantitative Transmural Phenotyping

Radiomics and AI are computational approaches designed to extract quantitative information from medical images beyond visual interpretation or conventional imaging scores. A typical workflow includes lesion segmentation, extraction of high-dimensional imaging features, feature selection, model development, and internal or external validation. In CD, these approaches have been applied to automated lesion segmentation, inflammatory activity assessment, treatment-response prediction and characterization of stricturing disease. Their clinical value depends not only on diagnostic performance, but also on reproducibility, interpretability, external validation and integration into clinically actionable decision pathways. Supplementary Table S5 summarizes recent original studies (Supplementary Materials).

For assessing inflammatory activity, MRE- and CTE-based models have shown promising performance. MRI radiomics may reduce reader-dependent variability in assessing terminal ileal inflammatory severity, performing comparably to the MaRIA score assessed by an experienced radiologist and better than the MaRIA score assessed by a junior radiologist [83]. On CTE, automated segmentation combined with radiomics-based classification may support more efficient and standardized activity assessment, although external validation remains limited [84].

Radiomics has also been explored for treatment-response prediction and healing assessment. CTE-based radiomics and deep-learning radiomics models have been developed to predict secondary loss of response to infliximab, early mucosal healing after biologic therapy, and stratified mucosal or transmural healing after infliximab treatment [85,86,87]. DECT radiomics may further integrate iodine-map and virtual monoenergetic information from the bowel wall and mesenteric fat, linking AI-based modeling with quantitative spectral imaging [88]. These applications are clinically attractive, but currently remain insufficiently validated for treatment selection or early drug switching.

The most clinically relevant application may be stricturing disease, where conventional imaging cannot reliably separate inflammation, fibrosis and muscular remodeling. CTE radiomics, MRE radiomics and IUS deep-learning approaches have shown potential for distinguishing inflammatory from fibrotic strictures and for improving upon visual interpretation in selected surgical cohorts [89,90,91,92]. Recent evidence also suggests that CTE radiomics may separately characterize inflammation, fibrosis and smooth muscle hyperplasia/hypertrophy, moving stricture phenotyping beyond the binary inflammation-versus-fibrosis framework [93]. However, most evidence comes from enriched surgical populations, and generalizability to routine clinical cohorts remains uncertain.

AI may additionally support workflow standardization by automating bowel-wall thickening detection on IUS or lesion segmentation on CTE [84,94]. These tools could help less experienced operators or central reading workflows, but they support image interpretation rather than validated, treatment-guiding decisions. 

Therefore, radiomics and AI expand quantitative transmural phenotyping through automated segmentation, activity assessment, response prediction and stricture characterization, but they remain research-level tools until externally validated and prospectively tested in clinical decision pathways.

## 9. Roadmap Toward Clinical Translation

Selected quantitative imaging biomarkers and study-specific thresholds for inflammatory activity, structural burden, and treatment response are summarized in Table 3. These values illustrate the potential clinical contribution of quantitative imaging while highlighting the heterogeneity of endpoints and the variable degree of validation across modalities. 

To facilitate clinical application of the proposed framework, a minimum structured imaging dataset could be organized according to the four phenotyping domains and adapted to the imaging modality. Reports should document the clinical indication, examination quality, and bowel segments assessed. For inflammatory activity, core parameters should include bowel wall thickness and color Doppler signal on IUS, and mural thickness, edema, ulceration, and enhancement-related features on MRE or CTE, with diffusion restriction additionally assessed on MRE when available. Structural burden should be characterized by stricture location and length, luminal narrowing, upstream dilation, and imaging features suggestive of inflammatory or fibrotic components. Extramural assessment should document mesenteric inflammatory changes, fistulas, abscesses, and other penetrating complications. Finally, longitudinal assessment should specify the timing of imaging relative to treatment initiation or optimization, changes in reproducible imaging measurements compared with baseline, and the evolution of structural or extramural abnormalities. Established imaging indices may complement these core descriptors, whereas CEUS, elastography, spectral CT parameters, and radiomics-derived features should currently be considered optional or investigational components. This proposed dataset is intended to facilitate standardized reporting rather than constitute a validated treatment-guiding algorithm.

Beyond technical validation, the implementation of quantitative transmural phenotyping must be considered in relation to healthcare resources, financial sustainability, and the availability of appropriately trained professionals. The economic burden varies across modalities and includes equipment acquisition and maintenance, examination and reporting time, contrast agents, specialized software, and personnel training. IUS offers a potentially cost-effective, radiation-free approach to longitudinal monitoring, although its implementation requires dedicated training and quality assurance. In a single-center UK service evaluation involving 193 patient care episodes, estimated costs were lower for IUS-based than for MRE-based management pathways (£78.86 vs. £375.35 per episode), with shorter intervals between referral and treatment initiation (46 vs. 91 days) [95]. However, these findings are context-specific and cannot be directly generalized to other healthcare systems. MRE provides comprehensive radiation-free assessment but requires greater scanner capacity, examination time, and specialist interpretation. Conventional CTE remains valuable for rapid assessment, particularly in acute settings, although cumulative radiation exposure limits its suitability for repeated monitoring, especially in younger patients. Advanced spectral techniques, including DECT and PCCT, as well as radiomics-based approaches, may involve further investments in equipment, software, technical expertise, and validation infrastructure.

Equally important, the availability of imaging equipment does not necessarily imply the availability of specialized services or professionals trained to acquire, interpret, and report quantitative CD imaging biomarkers. Access varies considerably across healthcare systems and geographical areas. For example, a recent Australian survey found that 82% of 122 responding gastroenterologists and trainees reported access to IUS, although availability was lower in non-metropolitan settings and shortages of funding and trained personnel remained important barriers [96]. Such survey findings cannot establish the total number of adequately equipped centers or qualified operators at national or international levels, and comparable workforce data remain limited. Therefore, the proposed framework should not be interpreted as requiring universal access to all imaging modalities. A pragmatic, resource-adapted approach would prioritize established monitoring techniques according to local availability and expertise, supported by structured training, multidisciplinary collaboration, standardized reporting, and referral pathways to specialized centers when necessary. Advanced quantitative and computational biomarkers should initially be concentrated in appropriately equipped centers and evaluated for their incremental clinical benefit and cost-effectiveness before broader implementation.

Translation of quantitative transmural phenotyping into clinical practice will require a staged approach. In the short term, international consensus should further refine and validate core imaging biomarkers, standardize acquisition and reporting procedures, and harmonize definitions of transmural response and healing across modalities, particularly IUS, MRE, and CTE. In the medium term, multicenter head-to-head studies should establish reproducibility, clinically meaningful thresholds, cross-platform generalizability, and incremental value over established clinical, biochemical, endoscopic, and histological measures. Key priorities include characterization of mixed inflammatory-fibrotic strictures, clarification of the prognostic relevance of residual mesenteric abnormalities, and external validation of radiomics and AI models. In the long term, prospective interventional trials should determine whether treatment adaptation based on imaging-derived targets improves patient-relevant outcomes, including relapse, hospitalization, bowel damage progression, and surgery. Only through this sequence of standardization, validation, and outcome-based testing can quantitative transmural phenotyping progress from complementary disease characterization to a validated component of precision management in CD.

## 10. Limitations of This Review

Several limitations should be considered when interpreting the findings of this review. First, the structured narrative approach was intended to provide a clinically relevant synthesis of imaging-derived biomarkers rather than an exhaustive systematic assessment of the literature. Although the PubMed search was complemented by screening the reference lists of relevant publications, the use of a single bibliographic database and the restriction to English-language studies may have resulted in the omission of pertinent evidence. Furthermore, the exclusion of pediatric populations limits the applicability of the findings to childhood-onset CD, in which radiation-free imaging is particularly relevant. As detailed records of exclusions were not maintained throughout the selection process, the reproducibility of study selection is also limited. 

Second, no formal risk-of-bias assessment was undertaken, and methodological appraisal relied on the characteristics and limitations reported in the original studies. Importantly, substantial heterogeneity in imaging protocols, quantitative biomarkers, measurement techniques, reference standards, definitions of transmural response and healing, and follow-up intervals limits direct comparisons across studies and modalities. Evidence supporting emerging approaches, particularly multienergy CT, elastography, radiomics, and AI, is also constrained by relatively small study populations, predominantly retrospective designs, and limited external validation. Potential publication and selective-reporting biases cannot be excluded. 

Despite these limitations, the proposed framework offers a clinically oriented interpretation of the available evidence and identifies key priorities for standardization, prospective validation, and the integration of quantitative transmural assessment into precision management of CD.

## 11. Conclusions

Quantitative transmural phenotyping provides a framework for extending CD assessment beyond the mucosa by integrating imaging-derived markers of mural inflammation, structural damage, mesenteric involvement, complications, and longitudinal response. IUS and MRE currently offer the most clinically mature approaches for repeated transmural monitoring, whereas conventional CTE remains particularly valuable for rapid assessment of structural burden, strictures, and penetrating complications. Multienergy CT, radiomics, and AI may further expand this framework through material-specific and computational biomarkers, but their routine clinical role remains limited by technical heterogeneity and insufficient external validation. At present, imaging-derived transmural phenotypes should complement, rather than replace, clinical, biochemical, endoscopic, and histological assessment. Their greatest potential lies in providing a more complete representation of disease burden and treatment response, thereby supporting increasingly individualized management of CD.

## Figures and Tables

**Table 1:** Imaging-derived biomarkers for quantitative transmural phenotyping in Crohn’s disease.

Modality	Main Imaging-Derived Markers	Main Clinical Contribution	Main Limitations
IUS	BWT, color Doppler signal, BWS, inflammatory mesenteric fat, lesion length, strictures, prestenotic dilation, complications	Repeatable bedside assessment of mural activity, extramural involvement, and early treatment response	Operator dependence, variable definitions of response and healing, limited assessment of proximal or deep pelvic segments
CEUS	Enhancement magnitude, time to peak, rise time, area under the time–intensity curve, wash-in/wash-out parameters	Dynamic assessment of bowel-wall perfusion associated with inflammatory activity	Protocol-, equipment-, ROI-, and software-dependence; limited evidence for distinguishing inflammation from fibrosis
Elastography	Strain ratio, shear-wave velocity, Young’s modulus	Quantification of bowel-wall stiffness, especially in stricturing disease	Stiffness is not fibrosis-specific and may reflect inflammation, edema, muscular remodeling, or bowel tension
MRE	BWT, edema, ulceration, enhancement, diffusion restriction, ADC, stricture length, upstream dilation, fistulas, abscesses, mesenteric changes	Comprehensive radiation-free transmural and extramural assessment; response monitoring and post-treatment risk stratification	Cost, availability, need for bowel preparation/distension, protocol heterogeneity, gadolinium and ADC standardization issues
Standard CTE	BWT, mural hyperenhancement, mural stratification, comb sign, mesenteric fat changes, strictures, upstream dilation, fistulas, abscesses	Rapid assessment of small-bowel disease, complications, and structural burden	Ionizing radiation, iodinated contrast, less suitable for repeated monitoring
Multienergy CT	ID, NID, virtual monoenergetic attenuation, spectral curve slope, dual-energy index, electron density, ECV	Quantitative tissue characterization, especially iodine-based assessment of inflammatory activity and mesenteric spectral phenotyping	Platform-, phase-, contrast-, and post-processing dependence; limited multicenter validation; PCCT evidence remains preliminary
Radiomics and AI	High-dimensional texture features, deep-learning features, automated segmentation, predictive model outputs	Automated lesion detection, activity assessment, response prediction, and granular stricture phenotyping	Mostly retrospective or internally validated models; limited interpretability, reproducibility, and clinical implementation

Abbreviations: IUS, intestinal ultrasound; BWT, bowel wall thickness; BWS, bowel wall stratification; CEUS, contrast-enhanced ultrasound; ROI, region of interest; MRE, magnetic resonance enterography; ADC, apparent diffusion coefficient; CTE, computed tomography enterography; CT, computed tomography; ID, iodine density; NID, normalized iodine density; ECV, extracellular volume fraction; PCCT, photon-counting computed tomography; AI, artificial intelligence.

**Table 2:** Main ultrasound and magnetic resonance enterography activity and response indices in Crohn’s disease.

Index	Modality	Components/Formula	Main Use	Main Limitations
SUS-CD [21]	IUS	Segmental categorical score based on BWT and color Doppler signal	Simple ultrasound activity scoring validated against endoscopic activity	Limited adoption compared with newer composite indices
BUSS [22]	IUS	BUSS = 0.75 × BWT + 1.65 × BWF	Quantitative BWT-flow model associated with endoscopic activity and prognosis	Does not include mesenteric fat or bowel wall stratification
IBUS-SAS [23]	IUS	IBUS-SAS = 4 × BWT + 15 × inflammatory fat + 7 × CDS + 4 × BWS	Composite mural and mesenteric activity score	Requires standardized acquisition and scoring of multiple parameters
MaRIA [24]	MRE	1.5 × BWT + 0.02 × RCE + 5 × edema + 10 × ulceration	Established MRE activity index for inflammatory severity and response assessment	Requires gadolinium and quantitative RCE measurement; relatively time-consuming
sMaRIA [25]	MRE	1 × BWT > 3 mm + 1 × mural edema + 1 × fat stranding + 2 × ulceration	Practical MRE activity and response index without quantitative enhancement measurement	Less extensively validated than MaRIA; ulcer detection may be reader-dependent
Clermont index [26]	MRE	−1.321 × ADC + 1.646 × BWT + 8.306 × ulceration + 5.613 × edema + 5.039	Diffusion-based MRE index incorporating ADC	ADC values are scanner- and protocol-dependent
C-score [27]	MRE	0.2 × (BWT − 3 mm) + 1.5 × enlarged lymph nodes + 2 × ulcer	Recent simplified MRI score for transmural healing and partial transmural response	Recently developed; requires further external validation before routine treatment-guiding use

Abbreviations: SUS-CD, Simple Ultrasound Score for Crohn’s Disease; IUS, intestinal ultrasound; BWT, bowel wall thickness; BUSS, Bowel Ultrasound Score; BWF, bowel wall flow; IBUS-SAS, International Bowel Ultrasound Segmental Activity Score; CDS, color Doppler signal; BWS, bowel wall stratification; MaRIA, Magnetic Resonance Index of Activity; MRE, magnetic resonance enterography; RCE, relative contrast enhancement; sMaRIA, simplified Magnetic Resonance Index of Activity; ADC, apparent diffusion coefficient; MRI, magnetic resonance imaging; C-score, modified Clermont score. BWF represents the binary assessment of color Doppler flow (absent/present) used in BUSS, whereas CDS represents the graded color Doppler signal (0–3) used in IBUS-SAS.

**Table 3:** Selected quantitative imaging biomarkers and study-specific thresholds for inflammatory activity, structural burden, and therapeutic response in Crohn’s disease.

Domain/Modality	Biomarker and Reported Threshold	Reference Standard/Endpoint	Reported Quantitative Result	Ref.
Inflammatory activity (MRE)	sMaRIA ≥ 1 (active); ≥ 2 (severe/ulcerative disease)	Segmental endoscopic activity and ulcers (CDEIS); retrospective development and independent responsiveness cohort.	Active: AUROC 0.91, sensitivity 90%, specificity 81%; severe: AUROC 0.94, sensitivity 85%, specificity 92%.	[25]
Inflammatory activity (MRE)	Segmental MaRIA ≥ 7 (active); ≥ 11 (severe/ulcerative disease)	Segmental endoscopic activity/ulcers; MaRIA comparison within the sMaRIA validation study.	Active: AUROC 0.89, sensitivity 81%, specificity 89%; severe: AUROC 0.97, sensitivity 95%, specificity 91%.	[25]
Inflammatory activity (DECTE)	Mean aorta-normalized mucosal iodine density: 20% [71]; 24.6% [72] (study-specific cut-offs)	Active inflammation: histopathology [71] versus terminal-ileal endoscopy [72]	Active inflammation: sensitivity 100%, specificity 75%, accuracy 92% [71]; AUROC 0.909, sensitivity 100%, specificity 81.8% [72].	[71,72]
Inflammatory activity (PCCTE)	Iodine density: 2.7 mg/mL (active); 3.4 mg/mL (moderate-to-severe vs. mild); study-specific cut-offs	Histopathological inflammation and severity; 23 patients.	Active: AUROC 1.00, sensitivity 97%, specificity 100%; severity: AUROC 0.85, sensitivity 83%, specificity 89%.	[75]
Transmural healing (IUS)	BWT ≤ 3 mm, commonly combined with normalization of other IUS activity features	Frequently applied sonographic definitions of TH, summarized in a systematic review.	Definition varies across studies; BWT alone does not establish complete TH.	[4]
Treatment response (IUS)	Postinduction BWT < 4.5 mm (weeks 14–26)	Subsequent TH (normalization of IUS parameters) at weeks 44–56; 129 infliximab-treated patients.	AUROC 0.85 for subsequent TH.	[32]
Treatment response (IUS)	IBUS-SAS < 25.0 after induction (weeks 14–26)	Mucosal healing (SES-CD ≤ 2) at weeks 44–56; 129 infliximab-treated patients.	AUROC 0.82 for subsequent mucosal healing.	[32]
Treatment response (IUS)	BUSS ≤ 3.52 at week 12	Endoscopic remission (SES-CD ≤ 2) at 12 months; 93 patients beginning biologics.	OR 9.93 for subsequent remission; sensitivity 59%, specificity 85%.	[31]
Transmural response (MRE)	C-score < 0.5 (TH); reduction ≥ 25% (TR25) or ≥ 50% (TR50)	Bowel damage progression; 1-year corticosteroid-free clinical/biochemical remission (DEVISE-CD).	TH: adjusted HR 0.15 (95% CI 0.04–0.53) for bowel damage progression in retrospective validation.	[27]
Post-treatment risk (MRE)	MaRIA ≥ 11 at week 46 after biologic initiation	Later surgery and clinical relapse during follow-up; 89 patients.	OR 11.6 for surgery; OR 4.4 for clinical relapse.	[48]
Structural burden (CTE)	Prestenotic dilation ≥ 2.5 cm as part of stricture assessment	Small-bowel stricture definition and subsequent adverse events; 1022 patients.	Dilation 2.5–3.0 cm vs. no stricture: adjusted HR 2.27 for small-bowel surgery.	[63]
Structural/extramural (CTE)	Mesenteric creeping fat index (MCFI) > 3	Moderate-to-severe fibrostenosis by surgical histopathology (prospective validation cohort).	AUROC 0.756 for fibrosis-related stricture severity.	[64]

Abbreviations: MRE, magnetic resonance enterography; sMaRIA, simplified Magnetic Resonance Index of Activity; CDEIS, Crohn’s Disease Endoscopic Index of Severity; AUROC, area under the receiver operating characteristic curve; MaRIA, Magnetic Resonance Index of Activity; DECTE, dual-energy computed tomography enterography; PCCTE, photon-counting computed tomography enterography; IUS, intestinal ultrasound; BWT, bowel wall thickness; TH, transmural healing; IBUS-SAS, International Bowel Ultrasound Segmental Activity Score; SES-CD, Simple Endoscopic Score for Crohn’s Disease; BUSS, Bowel Ultrasound Score; OR, odds ratio; TR25, ≥ 25% reduction in the C-score; TR50, ≥ 50% reduction in the C-score; HR, hazard ratio; CI; confidence interval; CTE, computed tomography enterography; MCFI, mesenteric creeping fat index. Note: This is a selective clinical summary, not a comparative ranking of modalities. Thresholds and performance estimates refer to study-specific populations, endpoints, reference standards, and acquisition methods and must not be used interchangeably or as universal treatment targets. Established use of IUS/MRE for monitoring does not imply that individual cut-offs are validated treatment targets. The BWT ≤3 mm threshold may be one component of a multimodal sonographic TH definition, not sufficient evidence of complete TH. Segmental MaRIA activity cut-offs are distinct from the prognostic application of MaRIA ≥ 11 at week 46. The aorta-normalized DECTE cut-offs of 20% [71] and 24.6% [72] derive from different clinical reference standards (histopathology and endoscopy, respectively) and are not interchangeable; additional published normalized iodine metrics differ by acquisition phase, normalization, and target endpoint [73,76]. Normalized iodine density (%) and absolute iodine density (mg/mL) are not directly comparable; spectral computed tomography thresholds remain preliminary. Contrast-enhanced ultrasound, elastography, and radiomics were not included because quantitative thresholds are not sufficiently standardized. Further examples of phase-dependent and endpoint-specific iodine thresholds are discussed in Section 7, with additional study-level details provided in Supplementary Table S4.

## Data Availability

No new data were created or analyzed in this study. Data sharing is not applicable to this article.

## Supplementary Materials

The following supporting information can be downloaded at: https://www.mdpi.com/article/10.3390/jcm15218696/s1, Search strategy for PubMed; Table S1: Main recent original ultrasound-based studies evaluating quantitative transmural phenotyping in Crohn’s disease; Table S2: Main recent original MRE-based studies evaluating quantitative transmural phenotyping in Crohn’s disease; Table S3: Main recent original studies evaluating conventional CTE in Crohn’s disease; Table S4: Main recent original studies evaluating multienergy CT in Crohn’s disease; Table S5: Main recent original studies evaluating radiomics and artificial intelligence for Crohn’s disease imaging.

## Abbreviations

CD	Crohn’s disease
IUS	intestinal ultrasound
MRE	magnetic resonance enterography
CTE	computed tomography enterography
CT	computed tomography
AI	artificial intelligence
CRP	C-reactive protein
FC	fecal calprotectin
CDEIS	Crohn’s Disease Endoscopic Index of Severity
SES-CD	Simple Endoscopic Score for Crohn’s Disease
DECT	dual-energy CT
SBCE	Small-bowel capsule endoscopy
DAE	device-assisted enteroscopy
PCCT	photon-counting CT
BWT	bowel wall thickness
BWS	bowel wall stratification
CEUS	contrast-enhanced ultrasound
ROI	region of interest
ADC	apparent diffusion coefficient
ID	iodine density
NID	normalized iodine density
NIC	normalized iodine concentration
ECV	extracellular volume fraction
ECCO	European Crohn’s and Colitis Organisation
SUS-CD	Simple Ultrasound Score for Crohn’s Disease
BUSS	Bowel Ultrasound Score
IBUS-SAS	International Bowel Ultrasound Segmental Activity Score
MaRIA	Magnetic Resonance Index of Activity
sMaRIA	simplified Magnetic Resonance Index of Activity
MRI	magnetic resonance imaging
DWI	diffusion-weighted imaging
MAGNIFI-CD	Magnetic Resonance Novel Index for Fistula Imaging in Crohn’s Disease
MCFI	mesenteric creeping fat index

## References

[1] Pariente B., Mary J.-Y., Danese S., Chowers Y., De Cruz P., D’Haens G., Loftus E.V., Louis E., Panés J., Schölmerich J. (2015). Development of the Lémann index to assess digestive tract damage in patients with Crohn’s disease. Gastroenterology.

[2] Kim M.C., Kim S.H., Koh S.J., Park J.H., Yoon H. (2022). Clinical implication of radiologic complete remission on Crohn’s Disease: Compared with endoscopic remission. Eur. J. Radiol..

[3] Oh K., Oh E.H., Noh S.M., Park S.H., Kim N., Hwang S.W., Park S.H., Yang D.-H., Byeon J.-S., Myung S.-J. (2022). Combined Endoscopic and Radiologic Healing Is Associated with a Better Prognosis Than Endoscopic Healing Only in Patients with Crohn’s Disease Receiving Anti-TNF Therapy. Clin. Transl. Gastroenterol..

[4] Geyl S., Guillo L., Laurent V., D’Amico F., Danese S., Peyrin-Biroulet L. (2021). Transmural healing as a therapeutic goal in Crohn’s disease: A systematic review. Lancet Gastroenterol. Hepatol..

[5] Kucharzik T., Tielbeek J., Carter D., Taylor S.A., Tolan D., Wilkens R., Bryant R.V., Hoeffel C., De Kock I., Maaser C. (2022). ECCO-ESGAR Topical Review on Optimizing Reporting for Cross-Sectional Imaging in Inflammatory Bowel Disease. J. Crohns Colitis.

[6] Yanai H., Feakins R., Allocca M., Burisch J., Ellul P., Iacucci M., Maaser C., Zilli A., Zidar N., Wilkens R. (2025). ECCO-ESGAR-ESP-IBUS Guideline on Diagnostics and Monitoring of Patients with Inflammatory Bowel Disease: Part 2: IBD scores and general principles and technical aspects. J. Crohns Colitis.

[7] Best W.R., Becktel J.M., Singleton J.W., Kern F. (1976). Development of a Crohn’s Disease Activity Index: National Cooperative Crohn’s Disease Study. Gastroenterology.

[8] Harvey R.F., Bradshaw J.M. (1980). A Simple Index of Crohn’s-Disease Activity. Lancet.

[9] Kucharzik T., Taylor S., Allocca M., Burisch J., Ellul P., Iacucci M., Maaser C., Baldin P., Bhatnagar G., Ben-Horin S. (2025). ECCO-ESGAR-ESP-IBUS Guideline on Diagnostics and Monitoring of Patients with Inflammatory Bowel Disease: Part 1. J. Crohns Colitis.

[10] Mary J.Y., Modigliani R. (1989). Development and validation of an endoscopic index of the severity for Crohn’s disease: A prospective multicentre study. Groupe d’Etudes Thérapeutiques des Affections Inflammatoires du Tube Digestif (GETAID). Gut.

[11] Daperno M., D’Haens G., Van Assche G., Baert F., Bulois P., Maunoury V., Sostegni R., Rocca R., Pera A., Gevers A. (2004). Development and validation of a new, simplified endoscopic activity score for Crohn’s disease: The SES-CD. Gastrointest. Endosc..

[12] Turner D., Ricciuto A., Lewis A., D’Amico F., Dhaliwal J., Griffiths A.M., Bettenworth D., Sandborn W.J., Sands B.E., Reinisch W. (2021). STRIDE-II: An Update on the Selecting Therapeutic Targets in Inflammatory Bowel Disease (STRIDE) Initiative of the International Organization for the Study of IBD (IOIBD): Determining Therapeutic Goals for Treat-to-Target strategies in IBD. Gastroenterology.

[13] Pennazio M., Rondonotti E., Despott E.J., Dray X., Keuchel M., Moreels T., Sanders D.S., Spada C., Carretero C., Cortegoso Valdivia P. (2023). Small-bowel capsule endoscopy and device-assisted enteroscopy for diagnosis and treatment of small-bowel disorders: European Society of Gastrointestinal Endoscopy (ESGE) Guideline—Update 2022. Endoscopy.

[14] Migaleddu V., Scanu A.M., Quaia E., Cossu Rocca P., Dore M.P., Scanu D., Azzali L., Virgilio G. (2009). Contrast-Enhanced Ultrasonographic Evaluation of Inflammatory Activity in Crohn’s Disease. Gastroenterology.

[15] Silverberg M.S., Satsangi J., Ahmad T., Arnott I.D.R., Bernstein C.N., Brant S.R., Caprilli R., Colombel J.-F., Gasche C., Geboes K. (2005). Toward an Integrated Clinical, Molecular and Serological Classification of Inflammatory Bowel Disease: Report of a Working Party of the 2005 Montreal World Congress of Gastroenterology. Can. J. Gastroenterol..

[16] Helwig U., Fischer I., Hammer L., Kolterer S., Rath S., Maaser C., Kucharzik T. (2022). Transmural Response and Transmural Healing Defined by Intestinal Ultrasound: New Potential Therapeutic Targets?. J. Crohns Colitis.

[17] Bae H., Seo N., Kang E.A., Cheon J.H., Lim J.S., Kim M.-J. (2023). Validation of the simplified magnetic resonance index of activity by using DWI without gadolinium enhancement to evaluate bowel inflammation in Crohn’s disease. Eur. Radiol..

[18] Dane B., Dillman J.R., Fidler J., Anupindi S.A., Fulmer C.G., Gordon I.O., Bruining D.H., Deepak P., Abualruz A.-R., Al-Hawary M. (2025). SAR Consensus Recommendations for Defining Small Bowel Crohn Disease Strictures at CT and MR Enterography. Radiology.

[19] Merola E., La Rocca D., Scaglione M., Pes G.M., Dore M.P. (2026). The missing evidence: Stricturing Crohn’s disease in clinical trials, a systematic review. Inflamm. Bowel Dis..

[20] Adamina M., Minozzi S., Warusavitarne J., Buskens C.J., Chaparro M., Verstockt B., Kopylov U., Yanai H., Vavricka S.R., Sigall-Boneh R. (2024). ECCO Guidelines on Therapeutics in Crohn’s Disease: Surgical Treatment. J. Crohns Colitis.

[21] Sævik F., Eriksen R., Eide G.E., Gilja O.H., Nylund K. (2021). Development and Validation of a Simple Ultrasound Activity Score for Crohn’s Disease. J. Crohns Colitis.

[22] Allocca M., Craviotto V., Bonovas S., Furfaro F., Zilli A., Peyrin-Biroulet L., Fiorino G., Danese S. (2022). Predictive Value of Bowel Ultrasound in Crohn’s Disease: A 12-Month Prospective Study. Clin. Gastroenterol. Hepatol..

[23] Novak K.L., Nylund K., Maaser C., Petersen F., Kucharzik T., Lu C., Allocca M., Maconi G., de Voogd F., Christensen B. (2021). Expert Consensus on Optimal Acquisition and Development of the International Bowel Ultrasound Segmental Activity Score [IBUS-SAS]: A Reliability and Inter-rater Variability Study on Intestinal Ultrasonography in Crohn’s Disease. J. Crohns Colitis.

[24] Rimola J., Rodriguez S., García-Bosch O., Ordás I., Ayala E., Aceituno M., Pellisé M., Ayuso C., Ricart E., Donoso L. (2009). Magnetic resonance for assessment of disease activity and severity in ileocolonic Crohn’s disease. Gut.

[25] Ordás I., Rimola J., Alfaro I., Rodríguez S., Castro-Poceiro J., Ramírez-Morros A., Gallego M., Giner À., Barastegui R., Fernández-Clotet A. (2019). Development and Validation of a Simplified Magnetic Resonance Index of Activity for Crohn’s Disease. Gastroenterology.

[26] Buisson A., Joubert A., Montoriol P.-F., Da Ines D., Hordonneau C., Pereira B., Garcier J.-M., Bommelaer G., Petitcolin V. (2013). Diffusion-weighted magnetic resonance imaging for detecting and assessing ileal inflammation in Crohn’s disease. Aliment. Pharmacol. Ther..

[27] Buisson A., Junda J., Vignette J., Lecoq E., Bouguen G., Goutorbe F., Scanzi J., Coban D., Dodel M., Bazoge M. (2024). Development and Validation of a Score to Assess Transmural Healing and Response in Patients with Crohn’s Disease. Clin. Gastroenterol. Hepatol..

[28] Calabrese E., Rispo A., Zorzi F., De Cristofaro E., Testa A., Costantino G., Viola A., Bezzio C., Ricci C., Prencipe S. (2022). Ultrasonography Tight Control and Monitoring in Crohn’s Disease During Different Biological Therapies: A Multicenter Study. Clin. Gastroenterol. Hepatol..

[29] Kucharzik T., Wilkens R., D’Agostino M.-A., Maconi G., Le Bars M., Lahaye M., Bravatà I., Nazar M., Ni L., Ercole E. (2023). Early Ultrasound Response and Progressive Transmural Remission After Treatment with Ustekinumab in Crohn’s Disease. Clin. Gastroenterol. Hepatol..

[30] Kucharzik T., Helwig U., Seibold F., Biedermann L., Högenauer C., Fischer I., Hammer L., Rath S., Maaser C., IBD DACH Study Group (2026). Early transmural response assessed by intestinal ultrasound predicts long-term clinical remission in patients with IBD: Results from the TRUST BEYOND study. Inflamm. Bowel Dis..

[31] Allocca M., Dell’Avalle C., Zilli A., Furfaro F., D’Amico F., Jairath V., Rubin D.T., Peyrin-Biroulet L., Fiorino G., Danese S. (2024). Ultrasound remission after biologic induction and long-term endoscopic remission in Crohn’s disease: A prospective cohort study. EClinicalMedicine.

[32] Huang Z., Cheng W., Chao K., Tang J., Li M., Guo Q., Liu G., Gao X. (2024). Baseline and Postinduction Intestinal Ultrasound Findings Predict Long-Term Transmural and Mucosal Healing in Patients with Crohn’s Disease. Inflamm. Bowel Dis..

[33] Vaughan R., Tjandra D., Patwardhan A., Mingos N., Gibson R., Boussioutas A., Ardalan Z., Al-Ani A., Gibson P.R., Christensen B. (2022). Toward transmural healing: Sonographic healing is associated with improved long-term outcomes in patients with Crohn’s disease. Aliment. Pharmacol. Ther..

[34] de Voogd F., Bots S., Gecse K., Gilja O.H., D’Haens G., Nylund K. (2022). Intestinal ultrasound early on in treatment follow-up predicts endoscopic response to anti-TNFα treatment in Crohn’s disease. J. Crohns Colitis.

[35] De Cristofaro E., Zorzi F., Colella A., Basile L., Castiglione F., Rispo A., Testa A., Guarino A.D., Lolli E., Cuccagna E. (2026). Early intestinal ultrasound assessment predicts therapy response: An easy tool for clinical decision-making. Inflamm. Bowel Dis..

[36] Cheng W., Huang Z., Qin S., Liang Z., Gao X., Liu G., Guo Q. (2024). Inflammatory mesenteric fat detected by Intestinal ultrasound is correlated with poor long-term clinical outcomes in patients with Crohn’s disease. Dig. Liver Dis..

[37] Ma L., He Y., Li W., Xiao M., Zhou M., Qin J., Yang H., Liu W., Zhu Q. (2024). Ultrasound Characteristics Can Predict Response to Biologics Therapy in Stricturing Crohn’s Disease. Clin. Transl. Gastroenterol..

[38] Ding S.-S., Liu C., Zhang Y.-F., Sun L.-P., Xiang L.-H., Liu H., Fang Y., Ren W.-W., Zhao H., Sun X.-M. (2022). Contrast-enhanced ultrasound in the assessment of Crohn’s disease activity: Comparison with computed tomography enterography. Radiol. Med..

[39] Servais L., Boschetti G., Meunier C., Gay C., Cotte E., François Y., Rozieres A., Fontaine J., Cuminal L., Chauvenet M. (2022). Intestinal Conventional Ultrasonography, Contrast-Enhanced Ultrasonography and Magnetic Resonance Enterography in Assessment of Crohn’s Disease Activity: A Comparison with Surgical Histopathology Analysis. Dig. Dis. Sci..

[40] Ainora M.E., Liguori A., Mignini I., Cintoni M., Galasso L., Laterza L., Lopetuso L.R., Garcovich M., Riccardi L., Gasbarrini A. (2024). Multimodal dynamic ultrasound approach as predictor of response in patients with Crohn’s disease treated with ustekinumab. Ther. Adv. Gastroenterol..

[41] Wilkens R., Liao D.-H., Gregersen H., Glerup H., Peters D.A., Buchard C., Tøttrup A., Krogh K. (2022). Biomechanical properties of strictures in Crohn’s disease: Can dynamic contrast-enhanced ultrasonography and magnetic resonance enterography predict stiffness?. Diagnostics.

[42] Mazza S., Conforti F.S., Forzenigo L.V., Piazza N., Bertè R., Costantino A., Fraquelli M., Coletta M., Rimola J., Vecchi M. (2022). Agreement between real-time elastography and delayed enhancement magnetic resonance enterography on quantifying bowel wall fibrosis in Crohn’s disease. Dig. Liver Dis..

[43] Matsumoto H., Hata J., Yo S., Sasahira M., Misawa H., Oosawa M., Handa O., Umegami E., Shiotani A. (2023). Serial changes in intestinal stenotic stiffness in patients with Crohn’s disease treated with biologics: A pilot study using ultrasound shear wave elastography. Turk. J. Gastroenterol..

[44] Chen Y.-J., He J.-S., Xiong S.-S., Li M.-Y., Chen S.-L., Chen B.-L., Qiu Y., Xia Q.-Q., He Y., Zeng Z.-R. (2024). Bowel Stiffness Assessed by Shear-Wave Ultrasound Elastography Predicts Disease Behavior Progression in Patients with Crohn’s Disease. Clin. Transl. Gastroenterol..

[45] Sun X., Wang C., Hu D., Ma G., Xu Z. (2025). Clinical value of multimodal ultrasound in evaluating intestinal stiffness and fibrosis in active Crohn’s disease. Insights Imaging.

[46] Hanžel J., Jairath V., Ma C., Guizzetti L., Zou G., Santillan C.S., Taylor S.A., van Viegen T., D’Haens G.R., Feagan B.G. (2022). Responsiveness of Magnetic Resonance Enterography Indices for Evaluation of Luminal Disease Activity in Crohn’s Disease. Clin. Gastroenterol. Hepatol..

[47] Revés J., Fernandez-Clotet A., Ordás I., Buisson A., Bazoge M., Hordonneau C., Ellul P., D’Anastasi M., Elorza A., Aduna M. (2025). Early Biological Therapy Within 12 Months of Diagnosis Leads to Higher Transmural Healing Rates in Crohn’s Disease. Clin. Gastroenterol. Hepatol..

[48] Fernández-Clotet A., Ordás I., Masamunt M.C., Caballol B., Rodríguez S., Gallego M., Barastegui R., Saavedra A.C., Panés J., Ricart E. (2024). Magnetic resonance enterography findings 46 weeks after initiation of biological therapy predict long-term adverse outcomes in Crohn’s disease. Aliment. Pharmacol. Ther..

[49] Rieder F., Baker M.E., Bruining D.H., Fidler J.L., Ehman E.C., Sheedy S.P., Heiken J.P., Ream J.M., Holmes D.R., Inoue A. (2024). Reliability of MR Enterography Features for Describing Fibrostenosing Crohn Disease. Radiology.

[50] Coimbra A., Rimola J., Cuatrecasas M., De Hertogh G., Van Assche G., Vanslembrouck R., Glerup H., Nielsen A.H., Hagemann-Madsen R., Bouhnik Y. (2022). Magnetic Resonance Enterography and Histology in Patients with Fibrostenotic Crohn’s Disease: A Multicenter Study. Clin. Transl. Gastroenterol..

[51] Lovett G.C., Schulberg J.D., Hamilton A.L., Wright E.K., Sutherland T.R., Ross A.L., Kamm M.A. (2025). Crohn’s Disease Stricture Response to Treatment Assessed with Magnetic Resonance Imaging and Intestinal Ultrasound: STRIDENT Randomized Trial. Inflamm. Bowel Dis..

[52] Kumar S., Plumb A., Mallett S., Clarke C., Parry T., Weng J.Y.J., Bhatnagar G., Bloom S., Hamlin J., Hart A. (2026). Magnetic resonance enterography to predict disabling disease in newly diagnosed Crohn’s disease: The METRIC-EF multivariable prediction model, multicentre diagnostic inception cohort study. Health Technol. Assess..

[53] Plumb A.A., Moran G., Chowdhury K., Ahmed N., Philpott S., Ahmad T., Bloom S., Hart A., Jacobs I., Menys A. (2025). Small Bowel Motility Quantified by Cine MRI to Predict Longer-Term Response in Patients with Crohn’s Disease Commencing Biological Therapy: The Motility Study. Inflamm. Bowel Dis..

[54] Hameed M., Taylor S.A., Ahmed N., Chowdhury K., Patel A., Helbren E., Bhagwanani A., Hyland R., Bhatnagar G., Sidhu H. (2025). Diffusion-weighted imaging to predict longer-term response in Crohn’s disease patients commencing biological therapy: Results from the MOTILITY trial. Br. J. Radiol..

[55] Brodersen J.B., Rafaelsen S.R., Nejatbakhsh E., Jensen M.D. (2026). Assessment of transmural treatment response in known Crohn’s disease: A prospective blinded study of the intermodality agreement between intestinal ultrasound and magnetic resonance enterography. BMJ Open Gastroenterol..

[56] Bhatnagar G., Mallett S., Beable R., Greenhalgh R., Ilangovan R., Lambie H., Mainta E., Patel U., Porté F., Sidhu H. (2024). Influence of diffusion weighted imaging and contrast enhanced T1 sequences on the diagnostic accuracy of magnetic resonance enterography for Crohn’s disease. Eur. J. Radiol..

[57] Hindryckx P., Jairath V., Zou G., Feagan B.G., Sandborn W.J., Stoker J., Khanna R., Stitt L., van Viegen T., Shackelton L.M. (2019). Development and Validation of a Magnetic Resonance Index for Assessing Fistulas in Patients with Crohn’s Disease. Gastroenterology.

[58] Beek K.J., Mulders L.G.M., van Rijn K.L., Horsthuis K., Tielbeek J.A.W., Buskens C.J., D’Haens G.R., Gecse K.B., Stoker J. (2025). External validation of the MAGNIFI-CD index in patients with complex perianal fistulising Crohn’s disease. Eur. Radiol..

[59] Anand E., Devi J., Ballard D.H., Joshi S., Tozer P., Hart A., Antoniou A., Deepak P., Stoker J., Lung P. (2026). Defining Radiological Healing in Perianal Fistulizing Crohn’s Disease: A TOpClass Global Expert Delphi Consensus. Clin. Gastroenterol. Hepatol..

[60] Ma L., Shen X., Chen Y.-J., Zhang Q., Li B., Zhang W. (2021). Computed tomography enterography for crohn’s disease: Correlation between the imaging findings and histopathologic scoring system. Abdom. Radiol..

[61] Tong J., Feng Q., Zhang C., Xu X., Ran Z. (2022). CT enterography for evaluation of disease activity in patients with ileocolonic Crohn’s disease. BMC Gastroenterol..

[62] Liu R., Liu S., Yi L., Wang D., Zhou X., Wang Z., Ren K., Ke J., Zhu W., Lu Y. (2023). Development and validation of multiparametric models based on computed tomography enterography to determine endoscopic activity and surgical risk in patients with Crohn’s disease: A multi-center study. Heliyon.

[63] Choi S.J., Choi E.S., Kim H.Y., Kim S., Jang J.K., Yoon Y.S., Ye B.D., Park S.H. (2026). Revised Criterion for Identifying Small-Bowel Stricture in Crohn Disease at CT Enterography. Radiology.

[64] Li X.-H., Feng S.-T., Cao Q.-H., Coffey J.C., Baker M.E., Huang L., Fang Z.-N., Qiu Y., Lu B.-L., Chen Z.-H. (2021). Degree of Creeping Fat Assessed by Computed Tomography Enterography is Associated with Intestinal Fibrotic Stricture in Patients with Crohn’s Disease: A Potentially Novel Mesenteric Creeping Fat Index. J. Crohns Colitis.

[65] Meng J., Mao Y., Zhou J., Chen Z., Huang S., Wang Y., Huang L., Zhang R., Shen X., Lv W. (2022). Mesenteric abnormalities play an important role in grading intestinal fibrosis in patients with Crohn’s disease: A computed tomography and clinical marker-based nomogram. Ther. Adv. Gastroenterol..

[66] Duan M., Guan B., Cao L., Zhou C., Huang W., Wu Q., Zhu W., Li Y. (2022). Computed tomography enterography predicts surgical-free survival in symptomatic stricturing Crohn’s disease. Abdom. Radiol..

[67] Fujimura H., Hashimoto S., Shimizu K., Onoda H., Tanabe M., Higashi M., Ogawa R., Goto A., Hamabe K., Nishikawa J. (2022). The Evaluation of Transmural Healing by Low-dose Computed Tomography Enterography in Patients with Crohn’s Disease. Intern. Med..

[68] Zhang F., Zhou J., Wang L., Zeng Z., Fu S., Xie P., Meng X. (2024). Residual abnormalities on CTE predict adverse outcomes in Crohn’s disease with endoscopic healing. Dig. Liver Dis..

[69] Zhang F., Guo M., Zhu P., Niu K., Zhou J., Wang L., Xie P., Shi L., Meng X. (2025). Creeping fat is associated with transmural healing in patients with Crohn’s disease receiving ustekinumab. Insights Imaging.

[70] Merola E., Mereu S., Dore M.P., Scaglione M. (2026). P0377 Dual-Energy and Photon-Counting Computed Tomography in Crohn’s Disease: Towards Quantitative Imaging Biomarkers. A Systematic Review. J. Crohns Colitis.

[71] Dane B., Sarkar S., Nazarian M., Galitzer H., O’Donnell T., Remzi F., Chang S., Megibow A. (2021). Crohn Disease Active Inflammation Assessment with Iodine Density from Dual-Energy CT Enterography: Comparison with Histopathologic Analysis. Radiology.

[72] Dane B., Kernizan A., O’Donnell T., Petrocelli R., Rabbenou W., Bhattacharya S., Chang S., Megibow A. (2022). Crohn’s disease active inflammation assessment with iodine density from dual-energy CT enterography: Comparison with endoscopy and conventional interpretation. Abdom. Radiol..

[73] Zhou H.-F., Chen W., Li J.-Q., Bai G.-J., Guo L.-L. (2024). Prediction of pathological activity in Crohn’s disease based on dual-energy CT enterography. Abdom. Radiol..

[74] Ni G., Su G., Shen H., Zhu P., Zhao H., Ao W. (2025). Optimization of dual-energy CT enterography parameters for the assessment of Crohn’s disease activity: A retrospective study. Abdom. Radiol..

[75] Dane B., Qian K., Soni R., Megibow A. (2024). Crohn’s disease inflammation severity assessment with iodine density from photon counting CT enterography: Comparison with endoscopic histopathology. Abdom. Radiol..

[76] Zhu C., Hu J., Rong C., Zhou J., Zhang B., Gao Y., Li C., Li J., Wu X. (2023). Mucosal healing assessment in Crohn’s disease with normalized iodine concentration from dual-energy CT enterography: Comparison with endoscopy. Insights Imaging.

[77] Dane B., Li X., Goldberg J.D., O’Donnell T., Le L., Megibow A. (2023). Crohn’s disease phenotype analysis with iodine density from dual-energy CT enterography. Abdom. Radiol..

[78] Li X., Wu W., Yuan Y., Zhu Z., Liu X., Xiao D., Long X. (2024). CT energy spectral parameters of creeping fat in Crohn’s disease and correlation with inflammatory activity. Insights Imaging.

[79] Lin Z., Hong M., Zhong H., Zheng X., Lin Y., Yang D., Zhong S., Yue X. (2025). Measurements on slope parameter mapping based on dual-energy CT enterography for improving Crohn’s disease diagnosis and inflammatory activity evaluation. Sci. Rep..

[80] Huang Q., Chen Z., Zhang R., Cai H., Yang X., Shen X., Huang L., Wang X., Zheng Q., Li M. (2025). Intestinal fibrosis assessment in Crohn’s disease patient using unenhanced spectral CT combined with 3D-printing technique. Insights Imaging.

[81] Gong M., Liu L., He P., Xu Y., Wu J., Yao H., Tan W., Zhang X., Li L., Li Y. (2026). Extracellular volume fraction derived from dual-energy CT characterizing the fibrosis of the bowel wall in stricturing Crohn disease. Jpn. J. Radiol..

[82] Sharifi A., O’Donnell T., Dane B. (2025). Optimizing photon counting CT enterography: Determining the optimal virtual monoenergy for bowel imaging. Abdom. Radiol..

[83] Ding H., Li J., Jiang K., Gao C., Lu L., Zhang H., Chen H., Gao X., Zhou K., Sun Z. (2022). Assessing the inflammatory severity of the terminal ileum in Crohn disease using radiomics based on MRI. BMC Med. Imaging.

[84] Gao Y., Zhang B., Zhao D., Li S., Rong C., Sun M., Wu X. (2024). Automatic Segmentation and Radiomics for Identification and Activity Assessment of CTE Lesions in Crohn’s Disease. Inflamm. Bowel Dis..

[85] Zhu C., Wang X., Wang S., Hu J., Gao Y., Li C., Li J., Wu X. (2023). Development and validation of a clinical radiomics nomogram to predict secondary loss of response to infliximab in Crohn’s disease patients. Heliyon.

[86] Zhu C., Liu K., Rong C., Wang C., Zheng X., Li S., Wang S., Hu J., Li J., Wu X. (2024). Computed tomography enterography-based deep learning radiomics to predict stratified healing in patients with Crohn’s disease: A multicenter study. Insights Imaging.

[87] Huang L., Li H., Wang S., Ren Y. (2025). Prediction of early mucosal healing of Crohn’s disease after treatment with biologics—A novel nomogram based on radiomics and clinical risk factors. Front. Pharmacol..

[88] Zhu C., Rong C., Song J., Zheng X., Wu Q., Hu J., Li J., Wu X. (2024). Evaluation of Mucosal Healing in Crohn’s Disease: Radiomics Models of Intestinal Wall and Mesenteric Fat Based on Dual-Energy CT. J. Imaging Inform. Med..

[89] Li X., Liang D., Meng J., Zhou J., Chen Z., Huang S., Lu B., Qiu Y., Baker M.E., Ye Z. (2021). Development and Validation of a Novel Computed-Tomography Enterography Radiomic Approach for Characterization of Intestinal Fibrosis in Crohn’s Disease. Gastroenterology.

[90] Meng J., Luo Z., Chen Z., Zhou J., Chen Z., Lu B., Zhang M., Wang Y., Yuan C., Shen X. (2022). Intestinal fibrosis classification in patients with Crohn’s disease using CT enterography-based deep learning: Comparisons with radiomics and radiologists. Eur. Radiol..

[91] Chirra P., Sleiman J., Gandhi N.S., Gordon I.O., Hariri M., Baker M., Ottichilo R., Bruining D.H., Kurowski J.A., Viswanath S.E. (2024). Radiomics to Detect Inflammation and Fibrosis on Magnetic Resonance Enterography in Stricturing Crohn’s Disease. J. Crohns Colitis.

[92] Zhou R., Shi J., Qin J., Xue X., Zhou W., Zhao X., He X., Ma L., Zhou M., Guo C. (2025). Comparison of Radiomics and Deep Learning Using Intestinal Ultrasound Images for Differentiating Inflammatory and Fibrotic Intestinal Strictures in Crohn’s Disease. J. Inflamm. Res..

[93] Sleiman J., Pillai V.G., Chirra P., Gandhi N.S., Gordon I.O., Hariri M., Baker M.E., Ream J., Fulmer C., El Ouali S. (2026). Development of a Computed Tomography Enterography-Based Radiomics Model to Characterize Inflammation, Fibrosis, and Smooth Muscle Thickening in Stricturing Crohn’s Disease. Am. J. Gastroenterol..

[94] Carter D., Albshesh A., Shimon C., Segal B., Yershov A., Kopylov U., Meyers A., Brzezinski R.Y., Ben Horin S., Hoffer O. (2023). Automatized Detection of Crohn’s Disease in Intestinal Ultrasound Using Convolutional Neural Network. Inflamm. Bowel Dis..

[95] Radford S.J., Abdul-Aema B., Tench C., Leighton P., Coad J., Moran G.W. (2024). Substantial cost savings of ultrasound-based management over magnetic resonance imaging-based management in an inflammatory bowel disease service. Scand. J. Gastroenterol..

[96] Chen L., Ruddick-Collins L., An Y.-K., Baraty B., Begun J., Boyapati R.K., Bryant R.V., Smith R.L., Srinivasan A.R. (2026). Examining attitudes to intestinal ultrasound in inflammatory bowel disease: A national survey of Australian gastroenterologists. Ther. Adv. Gastroenterol..

