# Supplementary material for: Beyond Mucosal Healing: Quantitative Transmural Phenotyping to Assess Therapeutic Response and Support Precision Treatment in Crohn’s Disease—A Narrative Review
Source: J Clin Med. 2026 Nov 7;15(21):8696. doi: 10.3390/jcm15218696 (PMC13763807; doi:10.3390/jcm15218696)
Supplement: Supplementary file 1 [file jcm-15-08696-s001.zip › jcm-4635831-supplementary.pdf]

Supplementary Materials

Search Strategy for PubMed

("Crohn Disease"[Mesh] OR Crohn*[tiab] OR "Crohn's disease"[tiab]) AND ("Diagnostic Imaging"[Mesh] OR "Ultrasonography"[Mesh] OR "Magnetic Resonance Imaging"[Mesh] OR "Tomography, X-Ray Computed"[Mesh] OR ultrasound[tiab] OR ultrasonography[tiab] OR "intestinal ultrasound"[tiab] OR "bowel ultrasound"[tiab] OR IUS[tiab] OR Doppler[tiab] OR "color Doppler"[tiab] OR "contrast-enhanced ultrasound"[tiab] OR CEUS[tiab] OR elastography[tiab] OR "shear wave elastography"[tiab] OR "strain elastography"[tiab] OR "magnetic resonance enterography"[tiab] OR MRE[tiab] OR "MR enterography"[tiab] OR "computed tomography enterography"[tiab] OR "CT enterography"[tiab] OR CTE[tiab] OR "dual-energy CT"[tiab] OR "dual energy CT"[tiab] OR DECT[tiab] OR "spectral CT"[tiab] OR "photon-counting CT"[tiab] OR "photon counting CT"[tiab] OR "iodine density"[tiab] OR "iodine concentration"[tiab] OR "iodine map"[tiab] OR "iodine maps"[tiab] OR radiomic*[tiab] OR "artificial intelligence"[tiab] OR "machine learning"[tiab] OR "deep learning"[tiab]) AND (transmural[tiab] OR mural[tiab] OR "bowel wall"[tiab] OR "intestinal wall"[tiab] OR "wall thickness"[tiab] OR "bowel wall thickness"[tiab] OR "mural thickness"[tiab] OR vascularity[tiab] OR hypervascularity[tiab] OR perfusion[tiab] OR enhancement[tiab] OR hyperenhancement[tiab] OR edema[tiab] OR oedema[tiab] OR "diffusion restriction"[tiab] OR "restricted diffusion"[tiab] OR "apparent diffusion coefficient"[tiab] OR ADC[tiab] OR fibrosis[tiab] OR fibrotic[tiab] OR stiffness[tiab] OR stricture*[tiab] OR stenosis[tiab] OR stenotic[tiab] OR fistula*[tiab] OR abscess*[tiab] OR penetrating[tiab] OR phlegmon[tiab] OR mesenteric[tiab] OR "creeping fat"[tiab] OR "fat wrapping"[tiab] OR "comb sign"[tiab] OR "transmural healing"[tiab] OR "transmural response"[tiab] OR "radiologic healing"[tiab] OR "radiological healing"[tiab] OR "radiologic remission"[tiab] OR "radiological remission"[tiab])

Table S1. Main recent original ultrasound-based studies evaluating quantitative transmural phenotyping in Crohn's disease

Study	Design and population	Reference/endpoint	Modality	Main outcome	Key findings	Clinical role
Calabrese et al., 2022 [28]	Prospective multicenter cohort; 188 CD patients treated with biologics	IUS-defined TH (normalization of all BUS parameters) and longitudinal changes during biological treatment	IUS	Treatment monitoring	TH at 12 months: 43/156 patients (27.5%)<br>Significant BWT improvement after biological therapy (p < 0.01)	Response assessment
Helwig et al., 2022 [16]	Post-hoc TRUST analysis; 351 IBD patients with increased BWT	IUS-defined TR/TH; clinical and sonographic outcome at week 52	IUS	TR/TH and outcome	TR 65.6% at wk 12; TH 23.9%-37.2%; early TH predicted favorable wk-52 sonographic outcome<br>Week-52 favorable sonographic outcome: OR 5.59 (95% CI 1.97–15.8) for early TH	Response and prediction
Kucharzik et al., 2023 [29]	Prospective exploratory IUS substudy of the STARDUST trial; 77 ustekinumab-treated CD patients (54 with week-48 IUS data)	IUS response (≥25% BWT reduction), transmural remission (normalization of IUS parameters), and week-48 endoscopic response (≥ 50% SES-CD reduction)	IUS	IUS response and transmural remission	At week 48, IUS response was observed in 25/54 patients (46.3%) and transmural remission in 13/54 (24.1%). Absence of IUS response at week 4 was associated with lack of terminal-ileal endoscopic response at week 48 (NPV 73%)	Early monitoring
Kucharzik et al., 2026 [30]	Prospective multicenter noninterventional TRUST BEYOND study; 163 IBD patients (74 CD, 89 UC) included in the 1-year analysis	IUS-defined TR/TH through week 52; week-52 clinical remission predicted by week-12 TR	IUS	TR/TH over 1 year	At week 52, TR and TH were achieved in 49/74 (66.2%) and 20/74 (27.0%) CD patients, respectively. In the overall IBD cohort, week-12 TR independently predicted clinical remission at week 52 (OR 4.3; 95% CI 2.45–7.52; p < 0.001)	Longitudinal monitoring

Study	Design and population	Reference/endpoint	Modality	Main outcome	Key findings	Clinical role
Allocca et al., 2022 [22]	Prospective cohort; 225 ileal/colonic CD patients	Endoscopic activity (SES-CD > 2) for BUSS derivation; 12-month negative clinical course (steroid use, treatment escalation, hospitalization, and/or surgery) for prognostic assessment	IUS, BUSS	12-mo negative disease course	BUSS >3.52 identified endoscopic activity (AUROC 0.864; sensitivity 83%; specificity 85%) and predicted a negative 12-month disease course (adjusted OR 6.97; 95% CI 2.87–16.93); baseline IUS complications also predicted adverse outcomes (adjusted OR 3.90)	Baseline risk stratification
Vaughan et al., 2022 [33]	Retrospective cohort; 202 CD patients in clinical remission	Sonographic healing, defined as BWT ≤ 3 mm without color Doppler hyperemia, inflammatory mesenteric fat, or disrupted bowel wall stratification. Clinical endpoints: medication escalation, corticosteroid use, hospitalization, and CD-related surgery during follow-up	IUS	Clinical outcomes	Sonographic healing was observed in 79/202 patients (39%). Compared with sonographic healing, persistent sonographic inflammation was independently associated with medication escalation (adjusted HR 1.94; 95% CI 1.23–3.06) and corticosteroid use (adjusted HR 2.41; 95% CI 1.24–4.67), but not with hospitalization or surgery	Prognosis in clinical remission
de Voogd et al., 2022 [34]	Prospective cohort; 40 anti-TNF-treated CD patients	Ileocolonoscopic response/remission after anti-TNF treatment	IUS, CEUS	Endoscopic response/remission	BWT reduction ≥18% at 4–8 weeks predicted endoscopic response (AUROC 0.765; sensitivity 82%; specificity 71%). At 12–34 weeks, ≥29% BWT reduction predicted response (AUROC 0.833; sensitivity 83%; specificity 88%); BWT ≤3.2 mm identified remission (AUROC 0.940; sensitivity 70%; specificity 85%). CEUS added limited value	Early response prediction
Allocca et al., 2024 [31]	Prospective cohort; 93 active CD patients starting biologics	Ileocolonoscopy-defined endoscopic remission (SES-CD ≤2) at 12 months	IUS, BUSS, IBUS-SAS	Endoscopic remission at 12 mo	Week-12 BUSS ≤3.52 predicted 12-month endoscopic remission (adjusted OR 9.93; sensitivity 59%; specificity 85%; NPV 87%). IBUS-SAS ≤22.8 identified endoscopic remission at final assessment (AUROC 0.906; sensitivity 95%; specificity 83%)	Early treatment target

Study	Design and population	Reference/endpoint	Modality	Main outcome	Key findings	Clinical role
Huang et al., 2024 [32]	Prospective infliximab cohort; 129 CD patients	IUS-defined TH (BWT ≤3 mm, preserved stratification, CDS ≤1, absent i-fat) and ileocolonoscopy-defined MH (total SES-CD ≤2) at weeks 44–56	IUS, IBUS-SAS	TH and MH	Postinduction BWT <4.5 mm predicted TH at weeks 44–56 (AUROC 0.85; sensitivity 85.7%; specificity 68.8%); IBUS-SAS <25.0 predicted overall MH (AUROC 0.82; sensitivity 75.8%; specificity 79.1%)	Prediction after induction
De Cristofaro et al., 2026 [35]	Prospective multicenter cohort; 142 CD patients starting biologics	12-month IUS response and transmural healing	IUS	TH and IUS response at 12 mo	At 12 months, TH occurred in 19% and IUS response in 44%. A BWT reduction of ≥1.25 mm at 3 months predicted 12-month TH (sensitivity 73%, specificity 61%) and IUS response (sensitivity 83%, specificity 57%). Combining ΔBWT with Limberg score improvement was associated with TH (OR 13.26; $p < 0.0001$) and IUS response (OR 20.9; $p < 0.001$)	Early decision support
Cheng et al., 2024 [36]	Retrospective cohort; 171 active CD patients treated with infliximab	Clinical remission, mucosal healing and transmural healing at 1 year	IUS	Impact of inflammatory mesenteric fat	Baseline inflammatory mesenteric fat was associated with lower 1-year transmural healing rates (21.6% vs. 51.7%; adjusted OR 0.364). Resolution of i-fat by week 14 was associated with improved 1-year clinical, mucosal, and transmural outcomes	Extramural prediction
Ma et al., 2024 [37]	Retrospective cohort; 43 stricturing CD patients treated with biologics	Surgery and clinical response in stricturing CD	IUS	Surgery and therapeutic outcomes	Creeping fat >180 degrees and high Limberg score predicted surgery; model AUROC 0.771; sensitivity 96.3%, specificity 62.5%	Stricturing disease prognosis
Ding et al., 2022 [38]	Retrospective study; 52 CD patients	SES-CD for intestinal inflammatory activity	CEUS vs CTE	Activity assessment vs SES-CD	BWT showed the best performance for detecting active CD: CEUS cut-off 6.5 mm (AUROC 0.887; sensitivity 90.5%; specificity 80%) vs. CTE cut-off 5.4 mm (AUROC 0.883; sensitivity 88.1%; specificity 90%)	CEUS activity assessment
Servais et al., 2022 [39]	Prospective preoperative cohort; 17 CD patients	Surgical histopathological inflammatory activity	US, CEUS, MRE	Histological activity	For severe histological inflammation, US BWT cut-off 7.5 mm (AUROC 0.85; sensitivity 84%; specificity 75%); CEUS time-to-peak cut-off 6.5 s (AUROC 0.88; sensitivity 84%; specificity 75%) and rise time cut-off 4.3 s (AUROC 0.86; sensitivity 84%; specificity 75%)	Perfusion and histological activity

Study	Design and population	Reference/endpoint	Modality	Main outcome	Key findings	Clinical role
Ainora et al., 2024 [40]	Prospective monocentric cohort; 52 anti-TNF-exposed patients treated with ustekinumab	Week-48 ileocolonoscopic response (≥ 50% reduction in segmental SES-CD); mucosal healing (SES-CD < 3)	IUS, CEUS, SWE	Endoscopic response at 1 year	Week-24 multimodal IUS score predicted endoscopic response with AUROC 0.91	Multimodal prediction
Wilkens et al., 2022 [41]	Prospective surgical cohort 25 enrolled; distensibility/biomechanical data available for 18; elastography feasible in 11/18.	Ex vivo biomechanical stiffness (Young's modulus) of resected bowel	US, CEUS, DCE-MRE, elastography	Ex vivo stricture stiffness	Stiffness associated with DCE-MRE enhancement slope, not CEUS; US prestenotic dilatation associated with stiffness<br>DCE-MRE enhancement slope vs stiffness: ϱ = 0.63 ($p = 0.007$); elastography vs stiffness: ϱ = −0.05 ($p = 0.87$)	Stricture biomechanics
Mazza et al., 2022 [42]	Cross-sectional study; 40 patients with ileal/ileocolonic CD. RTE-defined severe ileal fibrosis (strain ratio ≥2) was used as comparator for DE-MRE enhancement gain; no independent histological reference standard	Bowel-wall fibrosis assessment using elastography and delayed-enhancement MRE	Real-time elastography, DE-MRE	Fibrosis assessment	SR correlated with submucosal enhancement gain ($r = 0.594$; $p < 0.001$). DE-MRE %EG ≥21% identified RTE-defined severe fibrosis (AUROC 0.857; sensitivity 92%; specificity 82%). SR ≥2 was associated with adverse clinical outcomes (HR 5.4)	Fibrosis/stiffness assessment
Matsumoto et al., 2023 [43]	Pilot longitudinal study; 21 patients with stricturing CD: infliximab-naïve (n = 6), ustekinumab-naïve (n = 8), and infliximab-to-ustekinumab switch (n = 7)	Longitudinal shear-wave velocity after biological treatment	SWE	Serial stiffness changes	BWT decreased significantly after treatment in all three groups. Median shear-wave speed decreased significantly only in ustekinumab-naïve patients (n = 8), from 2.81 to 1.65 m/s at 1 year ($p = 0.028$), with no significant changes in the other treatment groups	Treatment monitoring in strictures
Chen et al., 2024 [44]	Retrospective cohort; 130 B1 CD patients	Progression from B1 to B2/B3 behavior during follow-up	SWE	Progression to B2/B3 disease	SWE independently predicted B1-to-B2/B3 progression (HR 1.08; 95% CI 1.03–1.12); proposed cut-off 12.75 kPa. Multivariable prognostic nomogram AUROC 0.792	Prognostic stratification

Study	Design and population	Reference/endpoint	Modality	Main outcome	Key findings	Clinical role
Sun et al., 2025 [45]	Single-center retrospective cohort; 63 CD patients (31 with intestinal stenosis, 32 without)	Endoscopy-based intestinal stenosis assessment (SES-CD used as reference by the authors); no histological validation of fibrosis	Multimodal US, SWE, IBUS-SAS	Stenosis detection	Shear-wave velocity AUROC 0.838; combined model AUROC 0.878; no histological validation. Overall ultrasound assessment of strictures vs endoscopy: sensitivity 71%, specificity 100%	Stenosis/stiffness assessment

Abbreviations: CD, Crohn's disease; IUS, intestinal ultrasound; TH, transmural healing; BUS, bowel ultrasonography; BWT, bowel wall thickness; IBD, inflammatory bowel disease; TR, transmural response; wk, weeks; OR, odds ratio; CI, confidence interval; SES-CD, Simple Endoscopic Score for Crohn's Disease; NPV, negative predictive value; UC, ulcerative colitis; BUSS, Bowel Ultrasound Score; mo, months; AUROC, area under the receiver operating characteristic curve; HR, hazard ratio; TNF, tumor necrosis factor; CEUS, contrast-enhanced ultrasound; IBUS-SAS, International Bowel Ultrasound Segmental Activity Score; CDS, color Doppler signal; i-fat, inflammatory mesenteric fat; MH, mucosal healing; ΔBWT, change in bowel wall thickness; CTE, computed tomography enterography; US, ultrasound; MRE, magnetic resonance enterography; SWE, shear-wave elastography; DCE-MRE, dynamic contrast-enhanced magnetic resonance enterography; ϱ, Spearman's rank correlation coefficient; RTE, real-time elastography; DE-MRE, delayed-enhancement magnetic resonance enterography; SR, strain ratio; r, correlation coefficient; %EG, percentage of enhancement gain; B1, non-stricturing non-penetrating behavior; B2, stricturing behavior; B3, penetrating behavior.

Table S2. Main recent original MRE-based studies evaluating quantitative transmural phenotyping in Crohn's disease

Study	Design and population	Reference/endpoint	MRE domain	Main findings	Clinical interpretation
Hanžel et al., 2022 [46]	Secondary analysis of two prospective observational studies; 41 active CD patients with paired baseline and week-12/14 MRE	Responsiveness of MRE activity indices using ≥ 0.5 SD improvement in radiologist-rated disease activity VAS (primary criterion); endoscopic CDEIS or CRP (secondary criteria)	Response assessment	sMaRIA showed the highest responsiveness (SES 1.17), followed by MaRIA (0.98), London extended index (0.95), and London index (0.85). sMaRIA was significantly more responsive than the London index, but not than MaRIA or the extended London index	MRE activity indices are responsive to treatment-related change.
Bae et al., 2023 [17]	Single-center retrospective diagnostic accuracy study; 55 CD patients (275 bowel segments) undergoing MRE and ileocolonoscopy within 2 weeks	Segmental ileocolonoscopic SES-CD assessment of inflammatory activity; contrast-enhanced sMaRIA and T2-only sMaRIA as imaging comparators	Non-contrast activity assessment	Modified non-contrast sMaRIA incorporating DWI grades instead of ulceration showed AUROC 0.863 for active inflammation, comparable to contrast-enhanced sMaRIA (0.908; $p = 0.122$) and superior to T2-only sMaRIA (0.827; $p = 0.017$)	DWI may support non-contrast MRE assessment in selected settings.
Revés et al., 2025 [47]	Multicenter retrospective cohort; 154 patients starting biologic therapy	MRE-defined TH (normalization of mural and extramural inflammatory abnormalities and absence of CD-related complications) assessed at 12 ± 6 months; subsequent bowel damage progression, CD-related surgery, hospitalization, and treatment escalation	MRE-defined TH (normalization of mural and extramural inflammatory abnormalities and absence of CD-related complications) assessed at 12 ± 6 months; subsequent bowel damage progression, CD-related surgery, hospitalization, and treatment escalation	Early biologic initiation (≤ 12 months) was associated with higher TH rates (32% vs. 14%; adjusted OR 3.23, 95% CI 1.36–7.70). TH was independently associated with reduced bowel damage progression (adjusted HR 0.28), CD-related surgery (0.21), and therapy escalation (0.35)	Association of early biologic initiation with TH and favorable long-term outcomes

Study	Design and population	Reference/endpoint	MRE domain	Main findings	Clinical interpretation
Buisson et al., 2024 [27]	DEVISE-CD project: retrospective development (n = 274), retrospective validation (n = 224), prospective validation (n = 46)	TH (C-score < 0.5), TR50 (≥50% C-score reduction), and TR25 (≥ 25% reduction). Endpoints: bowel damage progression in retrospective cohorts and 1-year corticosteroid-free clinical remission with fecal calprotectin <250 μg/g in the prospective cohort	Transmural response and healing	In the retrospective validation cohort, TH, TR50, and TR25 were associated with reduced bowel damage progression (adjusted HR 0.15, 0.36, and 0.46, respectively). In the prospective cohort, all three endpoints at week 12 predicted 1-year corticosteroid-free clinical and biochemical remission (OR 4.6, 6.9, and 6.0, respectively)	Both complete TH and partial TR showed prognostic relevance
Brodersen et al., 2026 [55]	Secondary analysis of a prospective, blinded, multicenter study; 35 CD patients with paired IUS and MRE at baseline and after 12 weeks of treatment	Intermodality agreement for transmural treatment response (IUS vs. MRE-based MaRIA and Clermont scores) and BWT normalization at 12 weeks; no independent reference standard for transmural response	Intermodality response assessment	IUS and MRE activity scores decreased after treatment. Intermodality agreement for transmural response was fair to moderate: IUS vs. MaRIA κ = 0.43; IUS vs. Clermont κ = 0.31). Agreement for BWT normalization was moderate (κ = 0.42)	IUS and MRE detect treatment-related changes but are not interchangeable for assessing transmural response
Fernández-Clotet et al., 2024 [48]	Prospective 2-year follow-up of 89 CD patients undergoing MRE reassessment at week 46 after initiation of biological therapy	Clinical relapse, CD-related surgery, endoscopic balloon dilation, hospitalization, and corticosteroid requirement during the subsequent 2-year follow-up	Post-treatment prognosis	Persistent severe inflammation (MaRIA ≥11) at week 46 predicted surgery (OR 11.6), surgery and/or endoscopic balloon dilation (OR 6.3), and clinical relapse (OR 4.6). Creeping fat predicted hospitalization (OR 5.1), while stricturing and/or penetrating complications predicted at least one adverse outcome (OR 3.35)	Persistent MRE abnormalities after biological therapy identify patients at increased risk of adverse clinical outcomes
Kumar et al., 2026 / METRIC-EF [52]	Multicenter inception cohort (nine UK hospitals); 194 newly diagnosed CD patients with prospective and retrospective follow-up data	Modified Beaugerie disabling CD within 5 years of diagnosis, excluding events occurring within the first 90 days	Baseline prognostic prediction	Disabling CD developed in 81/194 patients (41.8%). The clinical prediction model achieved an AUROC of 0.68. Adding baseline MEGS, sMaRIA, or Lémann Index did not improve prediction of disabling CD at 5 years compared with clinical predictors alone	Baseline MRE scores provided no additional prognostic value over clinical predictors for 5-year disabling disease

Study	Design and population	Reference/endpoint	MRE domain	Main findings	Clinical interpretation
Plumb et al., 2025 / MOTILITY [53]	Prospective multicenter cohort (13 centers); 199 biologic-treated patients with active non-stricturing small-bowel CD, of whom 86 completed the primary analysis	Response or remission at 1 year based on clinical and structural MRE criteria; postinduction cine-MRI motility changes assessed at weeks 12–30	Functional MRI prediction	Stable or improved postinduction motility MRI was more sensitive than CRP normalization for predicting 1-year response/remission (71.0% vs. 45.2%), but less specific (30.9% vs. 67.3%). Discrimination was poor and did not differ significantly (AUROC 0.48 vs. 0.53; $p = 0.65$)	Postinduction cine-MRI motility did not provide reliable discrimination for longer-term treatment response
Hameed et al., 2025 / MOTILITY DWI substudy [54]	Prespecified prospective multicenter DWI substudy of MOTILITY (6 centers); 25 patients with active small-bowel CD starting biologic therapy	Response/remission at 1 year defined using the MRE London Index or prespecified clinical failure criteria; no paired ileocolonoscopic validation	DWI prediction	Eight of 25 patients achieved 1-year response/remission. An ADC increase >10% showed sensitivity 37.5% and specificity 41.2%; a Clermont score reduction >25% showed sensitivity 37.5% and specificity 64.7%. Neither measure provided significant incremental prognostic value over CRP	Early DWI-derived changes showed limited prognostic performance in this small cohort
Rieder et al., 2024 [49]	Retrospective development (n = 99) and independent validation (n = 51) cohorts of patients with symptomatic terminal-ileal CD strictures	Interreader reliability of MRE stricture features (ICC) and association with radiologist-rated stricture severity (100-mm VAS); no independent histological reference standard	Stricturing disease and reliability	Stricture length showed the highest interreader reliability (ICC 0.85/0.91, development/validation), followed by maximal small-bowel dilation (0.74/0.73) and wall thickness (0.58/0.57). All three features were independently associated with radiologist-rated stricture severity ($p < 0.001$)	Reproducible MRE features may support standardized stricture assessment and trial endpoints
Coimbra et al., 2022 [50]	Prospective European multicenter surgical study (6 centers); 60 CD patients enrolled, 50 with evaluable histology-matched MRE (61 samples: 51 stenotic areas and 10 resection margins)	Full-thickness surgical histopathology using modified Chiorean fibrosis and inflammation scores (grades 0–3), assessed by two blinded central pathologists; moderate-to-severe fibrosis defined as grades 2–3 and active inflammation as grades ≥1	Fibrosis assessment	ADC and MaRIA correlated with histologic fibrosis ($r = -0.71$ and 0.59). FibrosisMRE detected moderate-to-severe histologic fibrosis (AUROC 0.910; cut-off ≥2.080; sensitivity 68.1%, specificity 100%). MaRIA detected active histologic inflammation (AUROC 0.966; cut-off ≥7.120; sensitivity 92.6%, specificity 100%)	Multiparametric MRE may help characterize fibrotic burden.

Study	Design and population	Reference/endpoint	MRE domain	Main findings	Clinical interpretation
Lovett et al., 2025 / STRIDENT [51]	Secondary imaging analysis of the STRIDENT randomized trial; 77 patients with stricturing CD allocated to intensive combination therapy (n = 52) or standard adalimumab monotherapy (n = 25)	Imaging-defined stricture improvement/resolution at 12 months on MRI and IUS; clinical response defined by improvement in obstructive symptoms	Stricture response	Clinical response occurred in 56/77 patients (73%). Complete stricture resolution was observed in 16/72 (22%) on MRI and 17/59 (29%) on IUS; additional stricture improvement occurred in 24/72 (33%) and 23/59 (39%), respectively	Serial imaging can document structural improvement and occasional resolution of CD strictures during medical treatment
Bhatnagar et al., 2024 [56]	Prespecified secondary analysis of the prospective multicenter METRIC study; 73 CD patients (28 newly diagnosed, 45 with suspected relapse)	Per-patient diagnostic accuracy for small-bowel CD against the METRIC outcome-based composite reference standard; comparison of T2/SSFP alone, with DWI, and with DWI plus contrast-enhanced T1 sequences	Protocol optimization	Sensitivity for small-bowel CD detection was 80%, 81%, and 79% across the three sequential protocols, with identical specificity (82%). Sensitivity for active disease was 97%, 97%, and 98%, respectively. Adding DWI or contrast-enhanced T1 sequences did not improve diagnostic performance	Supports investigation of simplified MRE protocols without routine additional sequences in selected diagnostic settings

Abbreviations: MRE, magnetic resonance enterography; CD, Crohn's disease; SD, standard deviation; VAS, visual analog scale; CDEIS, Crohn's Disease Endoscopic Index of Severity; CRP, C-reactive protein; sMaRIA, simplified Magnetic Resonance Index of Activity; SES, standardized effect size; MaRIA, Magnetic Resonance Index of Activity; SES-CD, Simple Endoscopic Score for Crohn's Disease; T2, T2-weighted imaging; DWI, diffusion-weighted imaging; AUROC, area under the receiver operating characteristic curve; TH, transmural healing; OR, odds ratio; CI, confidence interval; HR, hazard ratio; DEVISE-CD, project for developing and validating a transmural healing and response score in Crohn's disease; C-score, modified Clermont score; TR50, 50% transmural response; TR25, 25% transmural response; TR, transmural response; IUS, intestinal ultrasound; BWT, bowel wall thickness; κ, Cohen's kappa coefficient; METRIC-EF, Magnetic Resonance Enterography or Ultrasound in Crohn's Disease—Extended Follow-up; UK, United Kingdom; MEGS, Magnetic Resonance Enterography Global Score; MOTILITY, study of cine-MRI motility for treatment-response prediction in Crohn's disease; MRI, magnetic resonance imaging; ADC, apparent diffusion coefficient; ICC, intraclass correlation coefficient; r, correlation coefficient; FibrosisMRE, magnetic resonance enterography fibrosis score; STRIDENT, Stricture Definition and Treatment trial; METRIC, Magnetic Resonance Enterography or Ultrasound in Crohn's Disease study; SSFP, steady-state free precession; T1, T1-weighted imaging.

Table S3. Main recent original studies evaluating conventional CTE in Crohn's disease

Study	Design and population	Reference/endpoint	CTE domain	Main findings	Clinical interpretation
Ma et al., 2021 [60]	Retrospective imaging–histology correlation study; 34 CD patients undergoing CTE and endoscopic biopsy within 25 days.	Mucosal histopathological inflammatory activity assessed using a simplified Naini–Cortina scoring system.	Activity assessment and histopathologic correlation	Neutrophilic inflammation was associated with mural hyperenhancement, explaining 38.4% of its variance ($R^2 = 0.384$; $p = 0.006$). Lymphoplasmacytic inflammation was associated with larger mesenteric lymph nodes and increased mesenteric fat attenuation.	CTE features may reflect different components of mucosal inflammation but do not directly assess full-thickness histopathology.
Tong et al., 2022 [61]	Retrospective single-center study; 49 CD patients (280 ileocolonic segments) undergoing CTE and ileocolonoscopy within 24 hours.	Segmental ileocolonoscopic CDEIS and endoscopic lesion/ulceration assessment.	Activity scoring	Mural thickness, mural stratification, and comb sign independently predicted segmental CDEIS. CTEIA correlated with CDEIS (r = 0.779) and detected endoscopic lesions (AUROC 0.857; cut-off 6.25; sensitivity 77.5%; specificity 90.8%) and ulcerations (AUROC 0.901; cut-off 13.96; sensitivity 85.1%; specificity 83.0%).	CTEIA provides quantitative segmental assessment of ileocolonic inflammatory activity, requiring further validation.
Liu et al., 2023 [62]	Retrospective three-center study; 171 CD patients (256 bowel segments), with training (n = 121) and external validation (n = 50) cohorts.	Ileocolonoscopic SES-CD for segmental inflammatory activity (moderate-to-severe ≥7; severe ≥16); surgery-free survival during follow-up.	Activity scoring and surgical risk	Segmental CTE scores correlated significantly with SES-CD. Combined CTE–clinical nomograms achieved validation AUROCs of 0.837 for moderate-to-severe activity and 0.807 for severe activity. The CTE score was independently associated with surgery-free survival (HR 1.18; 95% CI 1.10–1.26).	CTE-based quantitative scores may contribute to endoscopic activity assessment and surgical risk stratification.
Choi et al., 2026 [63]	Retrospective cohort; 1022 CD patients without acute obstructive symptoms undergoing CTE during routine follow-up.	Prestenotic dilation thresholds (>3 cm vs. ≥2.5 cm) and subsequent emergency visits, small-bowel surgery, symptomatic obstruction, and penetrating complications.	Stricture definition and prognosis	Lowering the prestenotic dilation threshold from >3 to ≥2.5 cm increased stricture prevalence from 18.6% to 32.0% (137 additional patients). Compared with patients without strictures, those with 2.5–3.0 cm dilation had increased risks of emergency visits (adjusted HR 2.13), small-bowel surgery (2.27), symptomatic obstruction (7.99), and penetrating complications (3.47).	The revised prestenotic dilation threshold identifies additional patients with clinically relevant strictures and increased risk of adverse outcomes.

Study	Design and population	Reference/endpoint	CTE domain	Main findings	Clinical interpretation
Li et al., 2021 [64]	Retrospective development (n = 91) and prospective validation (n = 30) surgical CD cohorts undergoing preoperative CTE.	Surgical histological fibrosis grading (moderate-to-severe fibrosis ≥3 on a 0–4 scale); macroscopic fat wrapping assessed in the prospective cohort.	Creeping fat and fibrostenosis	MCFI correlated with histological fibrosis (r = 0.568/0.775, development/validation) and macroscopic fat wrapping (r = 0.840, validation). MCFI >3 discriminated moderate-to-severe histological fibrosis with AUROC 0.799/0.756, sensitivity 88.1%/92.3%, and specificity 50.0%/58.8%, respectively.	CTE-derived MCFI may serve as a quantitative mesenteric marker associated with fibrostenosing disease.
Meng et al., 2022 [65]	Retrospective three-center surgical cohort; 174 CD patients (training n = 91, internal test n = 30, external test n = 53) undergoing CTE within 3 months before surgery.	Full-thickness surgical histological fibrosis grading (Masson's trichrome; grades 0–4; moderate-to-severe fibrosis ≥3).	Mesenteric fibrosis model	A model combining MCFI, mesenteric edema, and disease duration discriminated moderate-to-severe histological fibrosis with AUROCs of 0.832, 0.821, and 0.850 in the training, internal test, and external test cohorts, respectively.	CTE-derived mesenteric features may contribute to fibrosis grading, although evidence remains limited to surgical cohorts.
Duan et al., 2022 [66]	Retrospective single-center study with temporal validation; 239 symptomatic stricturing CD patients (development n = 198; validation n = 41).	Surgical intervention versus successful nonsurgical management during follow-up; surgical histopathology available in a subset.	Stricturing disease and surgical risk	BWT <5.9 mm, residual luminal diameter >3.35 mm, and upstream bowel dilation <27.5 mm were independently associated with surgery-free survival. The combined nomogram achieved C-indexes of 0.905 and 0.892 in the development and validation cohorts, respectively.	Quantitative CTE measurements may support surgical risk stratification in symptomatic stricturing CD.
Fujimura et al., 2022 [67]	Retrospective single-center study; 122 CD patients undergoing low-dose CTE and ileocolonoscopy or balloon-assisted endoscopy within 2 weeks.	CTE-defined TH based on the absence of active mural and extramural abnormalities; endoscopic healing defined by the absence of ulcerative lesions.	Transmural healing assessment	CTE-defined TH occurred in 26/122 patients (21.3%), with substantial interreader agreement (κ = 0.743). Among 61 patients with endoscopic healing, only 22 (36.1%) also achieved TH. Overall CTE–endoscopy concordance was 56.5%.	CTE may detect residual transmural abnormalities despite endoscopic healing.
Zhang et al., 2024 [68]	Retrospective single-center cohort; 140 CD patients (217 bowel segments) in clinical remission (CDAI <150) and endoscopic healing, with CTE performed within 1 week of endoscopy.	Clinical relapse during follow-up, defined by CDAI >150, in patients with baseline endoscopic healing.	Residual disease and prognosis	Mesenteric edema, fibrofatty proliferation, and active small-bowel inflammation were associated with clinical relapse (univariable HRs 3.61, 3.40, and 2.74, respectively). A composite radiologic score ≥1 independently predicted clinical relapse (adjusted HR 3.21; 95% CI 1.63–6.31).	Residual CTE abnormalities despite endoscopic healing may identify patients at increased risk of clinical relapse.

Study	Design and population	Reference/endpoint	CTE domain	Main findings	Clinical interpretation
Zhang et al., 2025 [69]	Retrospective cohort; 113 active CD patients treated with ustekinumab, with baseline CTE assessment of mesenteric creeping fat.	Follow-up imaging-defined TH (CTE, MRE, or IUS), based on modality-specific criteria for normalization of bowel wall inflammation, mesenteric abnormalities, and complications.	Baseline mesenteric predictors of transmural healing	TH occurred in 26/113 patients (23.0%). Higher baseline MCFI (adjusted HR 0.67; 95% CI 0.49–0.91) and fibrofatty proliferation score (adjusted HR 0.50; 95% CI 0.29–0.85) were independently associated with a lower rate of TH during ustekinumab treatment.	Baseline CTE-derived mesenteric features may help predict subsequent transmural healing during biological therapy.

Abbreviations: CTE, computed tomography enterography; CD, Crohn's disease; R^2, coefficient of determination; CDEIS, Crohn's Disease Endoscopic Index of Severity; CTEIA, CT Enterography Index of Activity; r, correlation coefficient; AUROC, area under the receiver operating characteristic curve; SES-CD, Simple Endoscopic Score for Crohn's Disease; HR, hazard ratio; CI, confidence interval; MCFI, mesenteric creeping fat index; BWT, bowel wall thickness; C-index, concordance index; TH, transmural healing; κ, Cohen's kappa coefficient; CDAI, Crohn's Disease Activity Index; MRE, magnetic resonance enterography; IUS, intestinal ultrasound.

Table S4. Main recent original studies evaluating multienergy CT in Crohn's disease

Study	Design and population	Reference/endpoint	Technique / parameter	Main findings	Clinical interpretation
Dane et al., 2021 [71]	Retrospective study; 16 CD patients (25 bowel specimens: 19 surgical samples and 6 endoscopic biopsies).	Histopathological neutrophilic inflammatory activity (grade 0 vs. grades 1–3) in surgical specimens or terminal-ileal mucosal biopsies, assessed by a blinded pathologist.	DECTE; aorta-normalized mucosal iodine density	A study-specific aorta-normalized iodine density threshold of 20% identified active histological inflammation (17/25 segments; sensitivity 100%; specificity 75%; accuracy 92%).	Iodine density may quantify histologically active inflammation; findings are preliminary.
Dane et al., 2022 [72]	Retrospective study; 23 CD patients undergoing DECTE and ileocolonoscopy within 30 days.	Active terminal-ileal inflammation on endoscopy, defined by ileal SES-CD subscore ≥3 or Rutgeerts score >1.	DECTE; aorta-normalized and absolute terminal-ileal iodine density	An aorta-normalized iodine density threshold of 24.6% identified active endoscopic inflammation (AUROC 0.909; sensitivity 100%; specificity 81.8%). The absolute iodine density threshold of 1.78 mg/mL showed AUROC 0.750 (sensitivity 91.7%; specificity 63.6%).	Iodine-based quantification may support endoscopic activity assessment; larger validation cohorts are needed.
Zhu et al., 2023 [76]	Retrospective single-center cohort; 94 CD patients after infliximab (8 with TH, 34 with MH without TH, and 52 without MH); NIC analysis in 86 patients (34 MH vs. 52 non-MH).	Post-treatment endoscopic MH (SES-CD <3 and no mucosal ulcers) versus persistent ulcerative inflammation.	DECTE; normalized iodine concentration (NIC)	Mean NIC was lower with MH than without MH (0.337 vs. 0.556). A threshold of 0.448 discriminated MH from non-MH (AUROC 0.929; sensitivity 85.3%; specificity 82.7%; accuracy 83.7%). NIC plus fecal calprotectin achieved AUROC 0.964.	Iodine quantification may complement noninvasive assessment of endoscopic MH after infliximab.
Zhou et al., 2024 [73]	Retrospective study; 55 CD patients and 84 affected bowel segments (54 active, 30 inactive).	Histopathological active versus inactive inflammation, based on neutrophilic changes (e.g., cryptitis, crypt abscesses, erosions or ulcers).	DECTE; arterial- and venous-phase NIC, spectral-curve slope K, dual-energy index (DEI), and fat fraction	Arterial-phase NIC >0.245 identified histologically active segments (AUROC 0.908; sensitivity 83.3%; specificity 80.0%). Arterial-phase spectral-curve slope K and DEI showed AUROCs of 0.820 and 0.819; fat fraction did not differ significantly.	Spectral parameters may complement iodine-based quantification of pathological inflammatory activity.
Ni et al., 2025 [74]	Retrospective single-center study; 55 CD patients (23 remission–mild; 32 moderate–severe), with DECTE and ileocolonoscopy within 1 week.	Ileocolonoscopic CDEIS-defined activity: moderate–severe (≥10) versus remission–mild (<10).	DECTE; iodine concentration, wall enhancement, bowel wall thickness (BWT), and comb sign	A multiparametric DECTE model identified moderate-to-severe endoscopic activity (AUROC 0.933; sensitivity 90.6%; specificity 87.0%), outperforming iodine concentration (AUROC 0.855), BWT (0.908), and comb sign (0.719).	Combining spectral and morphological markers may improve endoscopic activity grading.

Study	Design and population	Reference/endpoint	Technique / parameter	Main findings	Clinical interpretation
Dane et al., 2024 [75]	Retrospective study; 23 CD patients undergoing photon-counting CT enterography with available pathological assessment.	Histopathological inflammatory activity and severity (22 endoscopic biopsies, 1 surgical specimen); supplementary clinical/biochemical assessment was used when pathology was temporally remote from imaging.	Photon-counting CT (PCCT); absolute iodine density	An iodine density threshold of 2.7 mg/mL identified active inflammation (AUROC 1.00; sensitivity 97%; specificity 100%; accuracy 98%). A 3.4 mg/mL threshold distinguished mild from moderate-to-severe active inflammation (AUROC 0.85; sensitivity 83%; specificity 89%; accuracy 87%); interreader ICC 0.94.	Promising, but preliminary, PCCT evidence for histological inflammation detection and severity assessment.
Dane et al., 2023 [77]	Retrospective study; 50 CD patients classified using imaging-defined small-bowel phenotypes.	Society of Abdominal Radiology–American Gastroenterological Association (SAR–AGA) morphologic phenotype classification, not histological fibrosis grading.	DECTE; absolute and aorta-normalized iodine density	Mean iodine density was 2.14, 3.54, 5.50, and 3.36 mg/mL for phenotype groups 1–2, 3–4, 5, and 6, respectively ($p = 0.001$). Aorta-normalized values were 21.20%, 39.47%, 40.98%, and 35.01% ($p < 0.001$); uptake was highest in strictures with active inflammation.	Iodine density differs across imaging phenotypes and appears more closely related to inflammation than to isolated fibrosis.
Li et al., 2024 [78]	Prospective single-center study; 40 surgical CD patients with confirmed creeping fat and 40 controls.	Surgically/pathologically confirmed creeping fat; clinical activity defined by CDAI ≥150 (active, n = 26) or <150 (inactive, n = 14).	DECTE; spectral characteristics of creeping fat	Enteric-phase spectral Hounsfield-unit curve slope (λHU) distinguished CDAI-defined active from inactive CD (cut-off 0.20 HU/keV; AUROC 0.930; sensitivity 92.3%; specificity 85.7%). Creeping-fat spectral parameters differed from normal mesenteric adipose tissue.	Spectral CT may characterize mesenteric involvement and its association with clinical activity.
Lin et al., 2025 [79]	Retrospective single-center study; 76 CD patients (167 bowel segments: 108 non-moderate-to-severe and 59 moderate-to-severe) and 53 controls.	CD diagnosis versus controls without intestinal disease; segmental endoscopic SES-CD (moderate-to-severe activity ≥7).	DECTE; slope parameter SP40–100 of bowel wall and adjacent mesenteric fat	Arterial-phase mesenteric-fat SP40–100 discriminated CD from controls (AUROC 0.969; cut-off −1.42; sensitivity 88.0%; specificity 98.0%). Arterial-phase creeping-fat SP40–100 discriminated moderate-to-severe endoscopic activity (AUROC 0.935; cut-off −0.99; sensitivity 79.7%; specificity 93.5%).	Mesenteric spectral slopes may provide additional information beyond conventional mural features.

Study	Design and population	Reference/endpoint	Technique / parameter	Main findings	Clinical interpretation
Huang et al., 2025 [80]	Single-center surgical proof-of-concept study; 12 CD patients and 85 histology-matched bowel specimens (6 patients/45 specimens for model development; 6/40 for prospective validation).	Full-thickness surgical histopathology (Masson's trichrome and H&E) aligned to spectral CT using 3D-printed molds; moderate-to-severe fibrosis grades 3–4 versus grades 0–2.	Unenhanced spectral CT; electron density (ED) and 50-keV monoenergetic attenuation	The combined model showed AUROC 0.828 (sensitivity 77.3%; specificity 82.6%) in development and 0.812 (sensitivity 63.6%; specificity 89.7%) in validation. Inflammation-adjusted AUROC increased numerically to 0.933, without a significant difference ($p = 0.168$).	Preliminary fibrosis-associated spectral biomarkers; the small surgical cohort limits generalizability.
Gong et al., 2026 [81]	Prospective preoperative surgical study; 22 CD patients with strictures and 66 histology-matched bowel segments.	Full-thickness surgical histopathology with separate grading of fibrosis and inflammatory activity.	DECT; extracellular volume (ECV) fraction and normalized iodine density	In segments with mild-to-moderate inflammation, mean ECV was higher in moderate-to-severe than in mild fibrosis (45.4% ± 5.7% vs. 38.2% ± 4.7%; $p < 0.001$), with AUROC 0.852. ECV did not significantly distinguish fibrosis grades in severely inflamed segments ($p = 0.45$).	ECV is associated with fibrosis, but its diagnostic performance depends on coexisting inflammation.

Abbreviations: CT, computed tomography; CD, Crohn's disease; DECTE, dual-energy computed tomography enterography; SES-CD, Simple Endoscopic Score for Crohn's Disease; AUROC, area under the receiver operating characteristic curve; TH, transmural healing; MH, mucosal healing; NIC, normalized iodine concentration; K, spectral-curve slope; DEI, dual-energy index; CDEIS, Crohn's Disease Endoscopic Index of Severity; BWT, bowel wall thickness; PCCT, photon-counting computed tomography; ICC, intraclass correlation coefficient; SAR–AGA, Society of Abdominal Radiology–American Gastroenterological Association; CDAI, Crohn's Disease Activity Index; λHU, slope of the spectral Hounsfield unit curve; HU, Hounsfield unit; keV, kiloelectronvolt; SP40–100, spectral slope parameter between 40 and 100 keV; H&E, hematoxylin and eosin; 3D, three-dimensional; ED, electron density; DECT, dual-energy computed tomography; ECV, extracellular volume.

Note: Reported thresholds are study-specific and should not be considered interchangeable or universally validated because of differences in acquisition protocols, normalization methods, reference standards, and clinical endpoints.

Table S5. Main recent original studies evaluating radiomics and artificial intelligence for Crohn's disease imaging

Study	Design and population	Reference/endpoint	Modality / approach	Main findings	Clinical interpretation
Li et al., 2021 [89]	Retrospective multicenter surgical cohort; 167 CD patients with 212 bowel lesions (98 training and 114 test lesions).	Full-thickness surgical histopathology (Masson's trichrome); none-to-mild fibrosis (grades 0–2) versus moderate-to-severe fibrosis (grades 3–4).	CTE radiomics	CTE radiomics discriminated moderate-to-severe histological fibrosis (AUROC 0.888 in training and 0.832 in the pooled test cohort). Center-specific test AUROCs were 0.816, 0.724, and 0.750, outperforming visual radiologist assessment (AUROC 0.554/0.598 in the pooled test cohort).	Early evidence that CTE texture features may capture fibrotic burden
Meng et al., 2022 [90]	Retrospective three-center surgical study; 235 CD patients (312 bowel segments), with training, internal test, and external test cohorts.	Full-thickness surgical histopathology (Masson's trichrome); moderate-to-severe fibrosis defined as grades 3–4 on a 0–4 scale.	CTE deep learning and radiomics	The deep-learning model identified moderate-to-severe histological fibrosis with AUROC 0.828 in training and 0.811 in the pooled test cohort (0.808 internal; 0.839 external). Performance was comparable to radiomics (AUROC 0.813) and superior to visual radiologist assessment (AUROC 0.579/0.646), with shorter processing time (48.4 vs. 599.8 s).	Deep learning may facilitate automated fibrosis characterization, although evidence remains limited to surgical cohorts.
Ding et al., 2022 [83]	Retrospective two-center study; 121 patients with terminal-ileal CD (91 for development/internal validation; 30 for external validation). MRI and ileocolonoscopy were performed within 3 days.	Terminal-ileal endoscopic inflammatory activity assessed using tCDEIS (ulcerative disease >7 vs. ≤7); senior- and junior-reader MaRIA scores served as imaging comparators.	MRI radiomics	MRI radiomics discriminated ulcerative disease with AUROC 0.872 in training, 0.824 in internal validation, and 0.800 in external validation. Performance was comparable to senior-reader MaRIA and superior to junior-reader MaRIA in the development/internal validation cohorts.	Radiomics may reduce reader-dependent variability in activity assessment
Zhu et al., 2023 [85]	Multicenter retrospective study; 181 CD patients treated with infliximab (training n = 102; testing n = 45; validation n = 34).	Secondary loss of response to infliximab at week 54 following an initial week-14 clinical response (HBI reduction >2), defined by absence of clinical remission (HBI ≥5) requiring treatment escalation or surgery.	CTE radiomics nomogram	Secondary loss of response occurred in 64/181 patients. A combined baseline CTE radiomics–HBI nomogram predicted secondary loss of response with AUROCs of 0.947 in training, 0.860 in testing, and 0.921 in validation.	Baseline CTE radiomics may contribute to risk stratification for secondary loss of response to infliximab.
Carter et al., 2023 [94]	Retrospective single-center IUS image-classification study; 308 CD patients contributing 1008 images (805 training; 203 held-out test images).	Expert-labeled IUS images classified as normal bowel wall (BWT ≤3 mm) versus thickened bowel wall (BWT >3 mm); no independent endoscopic or histopathological reference standard.	IUS convolutional neural network	The convolutional neural network discriminated normal from thickened bowel-wall images with accuracy 90.1%, reported sensitivity 86.4%, and reported specificity 94.0% in the 203-image test set (AUROC 0.978).	Proof of concept for automated IUS image interpretation, without evidence of improved patient-level clinical decisions.
Gao et al., 2024 [84]	Retrospective single-center study using two CTE datasets: 84 examinations for automated lesion segmentation and 193 examinations for radiomics-based activity classification.	Expert manual CTE segmentation for lesion delineation; ileocolonoscopic SES-CD for activity classification (mild 3–6 vs. moderate-to-severe ≥7).	CTE deep learning segmentation plus radiomics	nnU-Net achieved a Dice similarity coefficient of 0.824 for lesion segmentation. A radiomics-based logistic regression classifier discriminated moderate-to-severe endoscopic activity (AUROC 0.862; sensitivity 69.7%; specificity 84.0%; accuracy 75.9%).	Automated CTE segmentation and radiomics-based classification may facilitate imaging assessment, pending external validation.

Study	Design and population	Reference/endpoint	Modality / approach	Main findings	Clinical interpretation
Zhu et al., 2024 [88]	Retrospective two-center study; 106 CD patients with 221 diseased bowel segments (79 MH, 142 non-MH), divided into training (154 segments) and testing (67 segments).	Endoscopically defined segmental mucosal healing (MH), based on the absence of ulcerative lesions in the corresponding bowel segment.	DECTE iodine-map and virtual monoenergetic radiomics	Combined bowel-wall and mesenteric-fat iodine-map radiomics discriminated endoscopic MH from non-MH (AUROC 0.989 in training and 0.947 in testing; test sensitivity 97.7%, specificity 83.3%).	DECT iodine-map radiomics may integrate mural and mesenteric features for assessment of endoscopic mucosal healing.
Zhu et al., 2024 [86]	Retrospective multicenter study; 246 CD patients treated with infliximab (training n = 141; internal testing n = 61; external validation n = 44).	Post-treatment endoscopic MH (SES-CD <3 without ulcers); study-defined TH assessed by follow-up CTE or MRE in patients achieving MH.	CTE deep-learning radiomics	Baseline CTE deep-learning radiomics predicted subsequent MH (AUROC 0.948 in training, 0.889 in internal testing, and 0.938 in external validation). Among 86 patients with MH and available follow-up imaging, 21 achieved TH; the model discriminated TH with AUROC 0.856.	Baseline CTE radiomics may help predict mucosal healing and, preliminarily, subsequent transmural healing after infliximab.
Chirra et al., 2024 [91]	Retrospective single-center surgical study; 51 CD patients with terminal-ileal strictures and spatially matched MRE–histopathology (discovery n = 34; held-out validation n = 17).	Full-thickness surgical histopathology; severe inflammation and fibrosis independently defined by pathologist visual analog scores ≥70.	MRE radiomics	MRE radiomics discriminated severe histological inflammation (AUROC 0.69/0.67, discovery/validation) and fibrosis (AUROC 0.83/0.78). Adding radiologist visual scores improved inflammation discrimination to AUROC 0.79, whereas improvement for fibrosis was not significant.	MRE radiomics may provide complementary information on inflammatory and fibrotic components of CD strictures.
Huang et al., 2025 [87]	Single-center study; 154 CD patients treated with infliximab or ustekinumab. Retrospective development cohort (n = 120; training n = 84; internal validation n = 36) and prospective temporal validation cohort (n = 34).	Early endoscopic MH, defined as complete disappearance of baseline mucosal ulcers at 3–6 months after biological treatment.	CTE clinical-radiomics nomogram	A baseline CTE radiomics–clinical nomogram incorporating a radiomics score, BWT, and lymph-node diameter predicted early MH (AUROC 0.948 in training, 0.925 in internal validation, and 0.940 in prospective temporal validation).	Promising prediction of early mucosal healing after biological therapy, requiring independent external validation.
Zhou et al., 2025 [92]	Retrospective single-center surgical study; 64 CD patients and 87 preoperative IUS images assessed using five-fold cross-validation, without external validation.	Surgical histopathology with Masson's trichrome staining; fibrosis classification based on collagen-stained area above versus below the study-cohort median (40.10%).	IUS radiomics and deep learning	ResNet50 achieved 83.3% accuracy, 88.9% sensitivity, and 77.8% specificity for the study-defined fibrosis classification, compared with 67.0% accuracy for radiomics and 73.1% for expert assessment. No external validation was performed.	Preliminary evidence for AI-assisted IUS characterization of fibrotic burden in strictures, limited by the cohort-specific histological classification and small dataset.
Sleiman et al., 2026 [93]	Retrospective single-center surgical study; 100 CD patients with terminal-ileal strictures and spatially matched CTE–histopathology (discovery n = 66; held-out internal validation n = 34).	Full-thickness surgical histopathology separately grading inflammation, fibrosis, and smooth-muscle hyperplasia/hypertrophy using study-defined visual analog score thresholds (≥60, ≥40, and ≥50, respectively).	CTE radiomics	CTE radiomics distinguished severe histological inflammation, fibrosis, and smooth-muscle hyperplasia/hypertrophy with validation AUROCs of 0.71, 0.66, and 0.63, respectively. Corresponding validation accuracies were 74%, 68%, and 68%, exceeding those of visual radiologist assessment.	Proof of concept for separate characterization of inflammatory, fibrotic, and muscular components of CD strictures; limited discrimination and lack of external validation preclude routine clinical application.

Abbreviations: CD, Crohn's disease; CTE, computed tomography enterography; AUROC, area under the receiver operating characteristic curve; MRI, magnetic resonance imaging; tCDEIS, terminal-ileal Crohn's Disease Endoscopic Index of Severity; MaRIA, Magnetic Resonance Index of Activity; HBI, Harvey–Bradshaw Index; IUS, intestinal ultrasound; BWT, bowel wall thickness; SES-CD, Simple Endoscopic Score for Crohn's Disease; nnU-Net, no-new-Net; MH, mucosal healing; DECTE, dual-energy computed tomography enterography; DECT, dual-energy computed tomography; TH, transmural healing; MRE, magnetic resonance enterography; AI, artificial intelligence.
